# Drug resistance mechanism of kinase inhibitors in the treatment of hepatocellular carcinoma

**DOI:** 10.3389/fphar.2023.1097277

**Published:** 2023-02-13

**Authors:** Lei Jiang, Luan Li, Yongzhuang Liu, Meixiao Zhan, Ligong Lu, Shengtao Yuan, Yanyan Liu

**Affiliations:** ^1^ Guangdong Provincial Key Laboratory of Tumor Interventional Diagnosis and Treatment, Zhuhai People’s Hospital (Zhuhai Hospital AffiliatedWith Jinan University), Zhuhai, Guangdong, China; ^2^ Jiangsu Cancer Hospital, Jiangsu Institute of Cancer Research, The Affiliated Cancer Hospital of Nanjing Medical University, Nanjing, China; ^3^ Jiangsu Key Laboratory of Drug Screening, China Pharmaceutical University, Nanjing, Liaoning Province, China

**Keywords:** hepatocellular carcinoma, drug resistance, sorafenib, lenvatinib, regorafenib, cabozantinib

## Abstract

Hepatocellular carcinoma (HCC) is the most common form of primary liver cancer, and it usually occurs following chronic liver disease. Although some progress has been made in the treatment of HCC, the prognosis of patients with advanced HCC is not optimistic, mainly because of the inevitable development of drug resistance. Therefore, multi-target kinase inhibitors for the treatment of HCC, such as sorafenib, lenvatinib, cabozantinib, and regorafenib, produce small clinical benefits for patients with HCC. It is necessary to study the mechanism of kinase inhibitor resistance and explore possible solutions to overcome this resistance to improve clinical benefits. In this study, we reviewed the mechanisms of resistance to multi-target kinase inhibitors in HCC and discussed strategies that can be used to improve treatment outcomes.

## Introduction

Liver cancer is the second leading cause of cancer-related death worldwide, with approximately 850,000 new cases occurring annually, and hepatocellular carcinoma (HCC) accounts for approximately 90% of primary liver cancers. Hepatitis B and C virus infection, alcohol intake, ingestion of the fungal metabolite aflatoxin B1, and nonalcoholic steatohepatitis are potential risk factors for HCC (Llovet et al., 2021). Currently, patients with early HCC can be cured by radical hepatectomy, liver transplantation, and local ablation. Patients with intermediate HCC receive local therapy (e.g., chemoembolization), whereas patients with advanced HCC only benefit from systemic therapy (Llovet et al., 2018). With the in-depth study of targeted therapy for HCC, there is increasing evidence that multi-target combination therapy has a significant synergistic anti-tumor effect. Sorafenib, a multi-target kinase inhibitor with anti-angiogenic and anti-proliferative effects, prolongs the overall survival (OS) of patients with advanced HCC from 8 months to 11 months, and it was the only systemic therapeutic agent used to treat HCC between 2007 and 2016 (Llovet et al., 2008). In 2018, lenvatinib became the second first-line treatment approved for patients with advanced HCC (Kudo et al., 2018). Meanwhile, regorafenib was approved by the FDA in 2017 for second-line treatment in patients with unresectable HCC. In 2019, cabozantinib was used for second-line treatment in patients who had previously been treated with sorafenib (Bruix et al., 2017; Abou-Alfa et al., 2018).

Although systemic therapy for patients with advanced HCC can prolong the median survival time, it often leads to treatment failure because of the development of tumor cell resistance to kinase inhibitors, which has become a major obstacle to the clinical treatment of patients with advanced HCC. Development of resistance is the main reason for the poor prognosis of cancer as an incurable disease, and the emergence of resistance seems to be an inevitable consequence of tumor exposure to kinase-targeted therapy (Bagrodia et al., 2012). Resistance to existing therapies can be divided into two broad categories, including primary resistance and acquired resistance (Holohan et al., 2013). Primary resistance occurs at the beginning of drug treatment; that is, genetic heterogeneity of tumor cells leads to insensitivity to therapeutic drugs. Conversely, acquired resistance describes resistance in which, after a period of clinical benefit, kinase inhibitors gradually become ineffective during treatment. (Bagrodia et al., 2012; Jadidi-Niaragh et al., 2016). The most common cause of acquired resistance is the activation of signaling pathways that bypass drug targets to maintain survival and proliferation. In fact, because kinase inhibitors generally target a variety of signaling pathways, resistance occurs once the related compensatory cascade signaling pathways are activated. At the same time, a large number of new targets have also been found to be associated with HCC resistance (Tang et al., 2020). Determining the resistance factors of kinase inhibitors and exploring the regimens that can be used to overcome or delay drug resistance has certain guiding significance for clinical treatment. Therefore, in this review, we summarized the results of recent studies on the potential mechanisms leading to resistance to kinase inhibitors and explored some strategies that could be used to improve treatment outcomes.

## Drug resistance of sorafenib

In 2007, sorafenib, a small-molecule targeted drug, was approved for the treatment of advanced liver cancer. In the following decade, sorafenib remained the only first-line targeted therapy for advanced HCC (Llovet et al., 2008). As an oral receptor tyrosine kinase inhibitor, it inhibits intracellular serine/threonine kinases (including the Raf/MEK/ERK signaling pathway) and receptor tyrosine kinases [RTKs; including vascular endothelial growth factor receptors (VEGFR-1), VEGFR-2, VEGFR-3, platelet-derived growth factor receptor (PDGFR)-β, c-KIT, FMS-like tyrosine kinase 3 (FLT-3), and rearranged during transfection (RET)], thereby inhibiting tumor growth and angiogenesis (Tang et al., 2020). Although patients with HCC who received sorafenib exhibited a significant increase in mean OS, only a small number of patients obtained a real and long-term benefit from this therapy (Keating, 2017). Thus, elucidating the mechanism of sorafenib resistance is important for prolonging the survival of patients with HCC.

## Primary drug resistance

### Tumor heterogeneity and EGFR

Genomic instability, from single-base substitutions to doubling of the whole genome, provides raw materials for generating tumor heterogeneity and is essential for the development and progression of many cancers^[12,13]^. Currently, some researchers believe that tumour heterogeneity can be broadly divided into intertumoural and intratumoural heterogeneity (Dagogo-Jack and Shaw, 2018). During tumor progression, intratumoral heterogeneity is maintained by selective pressures that include exogenous exposures, internal environmental dynamics and cancer therapies themselves (Swanton, 2017). The process of selective therapeutic pressure to maintain intratumoral heterogeneity can be described as the disappearance of targeted cell clones, the acquisition of new resistance mutations, signaling and epigenetic adaptive responses and finally complete alteration of the tumor phenotype (Vasan et al., 2019). Maintaining intratumoral heterogeneity drives the ability of cancer cells to adapt to stressful conditions including chemotherapy (Swanton, 2017). Under therapeutic pressure, tumor cells become the basis for the development of chemoresistance by changing the dose of specific gene products, such as therapeutic targets, drug efflux pumps, or metabolic enzymes (Ippolito et al., 2021). For kinase-targeted agents, however, intertumoural heterogeneity (heterogeneity between patients with tumors of the same histological type) determines whether tumor patients exhibit primary resistance (O'Connor et al., 2007). In HCC, this heterogeneity is reflected in the overexpression and aberrant activation of EGFR in some patients (Ito et al., 2001). EGFR is a 170-kDa transmembrane glycoprotein composed of an extracellular domain that recognizes and binds specific ligands as well as an intracellular domain that acts as a protein kinase. Activated EGFR stimulates the activation of several signal transduction pathways (Dagogo-Jack and Shaw, 2018). The expression and activation of EGFR and its major dimerization partner HER-3 (ErbB-3) are frequently dysregulated in HCC (Negrini et al., 2010). Blivet-Van Eggelpoël et al. provided evidence that dysregulation of the EGFR/HER-3 signaling pathway limits the efficacy of sorafenib in treatment-naïve or acquired-resistant HCC cells. Therefore, anti-EGFR therapy might improve the therapeutic benefit of sorafenib by alleviating primary resistance (Ippolito et al., 2021). Investigators have used several different methods to block the expression of EGFR, the kinase activity of EGFR, or its autocrine activation, thereby increasing the sensitivity of EGFR-positive resistant cells to sorafenib, further demonstrating that EGFR is a potential determinant of sorafenib resistance in HCC cells. Therefore, biological analysis of EGFR, whether directly measuring EGFR expression or activity or detecting its ligands, will help predict the efficacy of sorafenib, which will be a promising personalized treatment option for patients with _HCC_ (O'Connor et al., 2007).

### Cancer stem cells (CSCs)

In many solid tumors, a small proportion of cells with progenitor-like features called CSCs or tumor-initiating cells are present (Ito et al., 2001). Accumulating evidence suggests that CSCs are involved in tumor recurrence, metastasis, and chemoresistance, leading to tumor progression and patient death. Tovar et al. found that tumor tissues from patients with sorafenib-resistant HCC have a higher proportion of CSCs (Tovar et al., 2017). Xin et al. conducted a study to test the hypothesis that hepatocarcinoma-derived CSCs are resistant to sorafenib treatment (Xin et al., 2013). The increase in CSC counts was accompanied by reduced apoptosis compared with the findings in the presence of non-CSCs, and the reduction in apoptosis was associated with excessive activation of AKT and ERK. In this study, CSCs were linked to a survival advantage over non-CSCs after sorafenib treatment of tumor cells, resulting in a significant increase in the relative proportion of CSCs in all HCC cell lines tested, and this may be related to liver cancer recurrence after sorafenib treatment (Xin et al., 2013). In addition, Etienne Ho Kit Mok et al. showed that SREBP2-mediated cholesterol biosynthesis is crucial for the increase of hepatic CSCs, and deletion of sterol-regulatory element binding protein 2 (SREBP2) and its chaperone SCAP conferred sensitivity to tyrosine kinase inhibitors in tumor-bearing _mice_ (Li et al., 2022; Mok et al., 2022).

**FIGURE 1:**
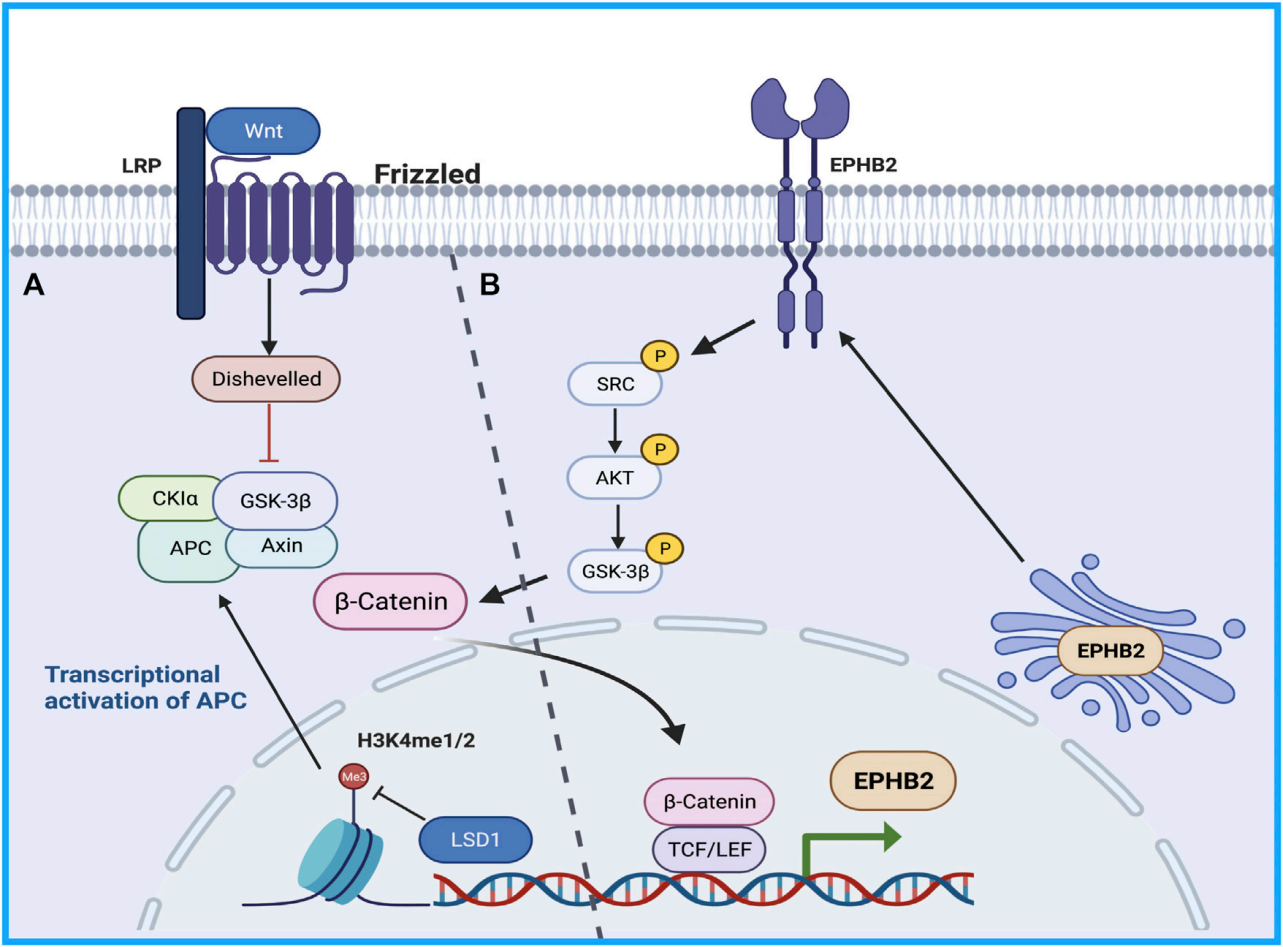
The Wnt/β-catenin pathway regulates stem cell proliferation and differentiation. **(A)** LSD1 is able to demethylate the monomethyl and dimethyl residues of lysine-4 on histone H3, thereby inhibiting the expression of several suppressors of β-catenin signaling promoting β-catenin activation, thereby promoting self-renewal and drug resistance in CSCs. **(B)** TCF1/EPHB2/β-catenin positive feedback loop regulates cancer stemness and drug resistance.

Therefore, the study of the mechanism of drug resistance in CSCs will provide good clinical benefits in sorafenib-resistant patients. The Wnt/β -catenin pathway regulates stem cell proliferation and differentiation, and the Wnt signaling pathway is closely related to drug resistance caused by CSCs and tumor recurrence and metastasis (Lou and Dean, 2007). Histone demethylation has proven essential for the self-renewal/differentiation of stem cells, and the activity of lysine-specific demethylase 1 (LSD1) is required for the appearance of CSCs after long-term sorafenib treatment in patients with HCC. LSD1 can demethylate the monomethyl and dimethyl residues of lysine-4 (H3K4me1 or H3K4me2) on histone H3, thereby inhibiting the expression of several suppressors of β-catenin signaling, especially Prickle 1 and APC in Lgr5+CSCs, and promoting the activation of β-catenin, thereby stimulating self-renewal and drug resistance in Lgr5+CSCs (Lei et al., 2015). Studies illustrated that LSD1 inhibitors can partially restore the sensitivity of resistant cells to sorafenib by inhibiting the Wnt/β-catenin signaling pathway and reducing the self-renewal capacity of CSCs (Huang et al., 2017). In addition, some researchers found that EPHB2 kinase expression is elevated in sorafenib-resistant HCC cells, and this kinase regulates cancer stemness and drug resistance through the TCF1/EPHB2/β-catenin positive feedback loop. In immunocompetent mouse models, targeting EPHB2 with rAAV-8-shEPHB2 (EPHB2 inhibitor) inhibited HCC tumor growth and sensitized HCC cells to sorafenib (Leung et al., 2021), indicating that targeting tumor cell stemness may be a feasible therapeutic strategy against sorafenib resistance in HCC (Figure 1).

## Acquired drug resistance

### EGFR and HGF/cMet mediated signaling pathway

Dysregulation of the Ras-Raf-Mek-ERK, PI3K-Akt-mTOR, PLC- γ 1, signal transducer and activator of transcription, and Src pathways downstream of EGFR are involved in tumor cell proliferation and apoptosis, which are tightly associated with sorafenib resistance (Swanton, 2017). Since 2007, preclinical studies have elucidated that many growth factors, including hepatocyte growth factor, insulin-like growth factor, and fibroblast growth factor, play vital roles in sorafenib resistance by activating the PI3K/AKT and Ras/Raf/MEK/ERK pathways (Arao et al., 2013; Nishida et al., 2015; Han et al., 2017; Vakil and Trappe, 2021). The PI3K/AKT/mTOR signaling pathway is one of the most common dysregulated pathways in human cancer, and it controls key cellular processes, such as metabolism, motility, growth, and proliferation (Janku et al., 2018). Previous studies illustrated that acquired resistance to sorafenib in HCC may be caused by compensatory activation of the PI3K/AKT pathway. AKT activation promotes tumor cell proliferation and apoptosis resistance; thus, inhibition of AKT activity reverses acquired resistance to sorafenib in HCC (Chen et al., 2011). Similarly, such compensatory signaling activation included the Ras/Raf/MEK/ERK pathway, and MAPK levels influence HCC sensitivity to sorafenib. If only the ERK cascade or AKT pathway is activated after sorafenib treatment, cancer cells could evade apoptosis (Aksamitiene et al., 2012). Recently, it has been reported that c-Jun N-terminal kinase (JNK), a member of the MAPK family, can be used as a biomarker to predict sorafenib sensitivity (Vasan et al., 2019).

Han et al. found using sorafenib-resistant HCC cells generated from sorafenib-sensitive human HCC cells that continuous exposure to sorafenib increased hepatocyte growth factor production and c-Met phosphorylation, leading to the activation of AKT and ERK pathways (Han et al., 2017). Dual inhibition of Akt and cMET by the inhibitors MK2206 and capmatinib, respectively, could inhibit the proliferation of sorafenib-resistant HCC cells *in vitro* and sorafenib-resistant HCC xenografts in mice (Han et al., 2017). Tivantinib, a highly selective inhibitor of cMET, has demonstrated its value in different tumors (Santoro et al., 2013; Calles et al., 2015; Moosavi et al., 2021). It has been shown that by inhibiting the expression of EMT and MDR (multidrug resistance) related genes, the combination of tivatinib slowed the clearance of sorafenib in HCC cells and enhanced the anti-tumor effect of sorafenib (Aoyama et al., 2014; Gao et al., 2019).

## Glycolysis

**FIGURE 2:**
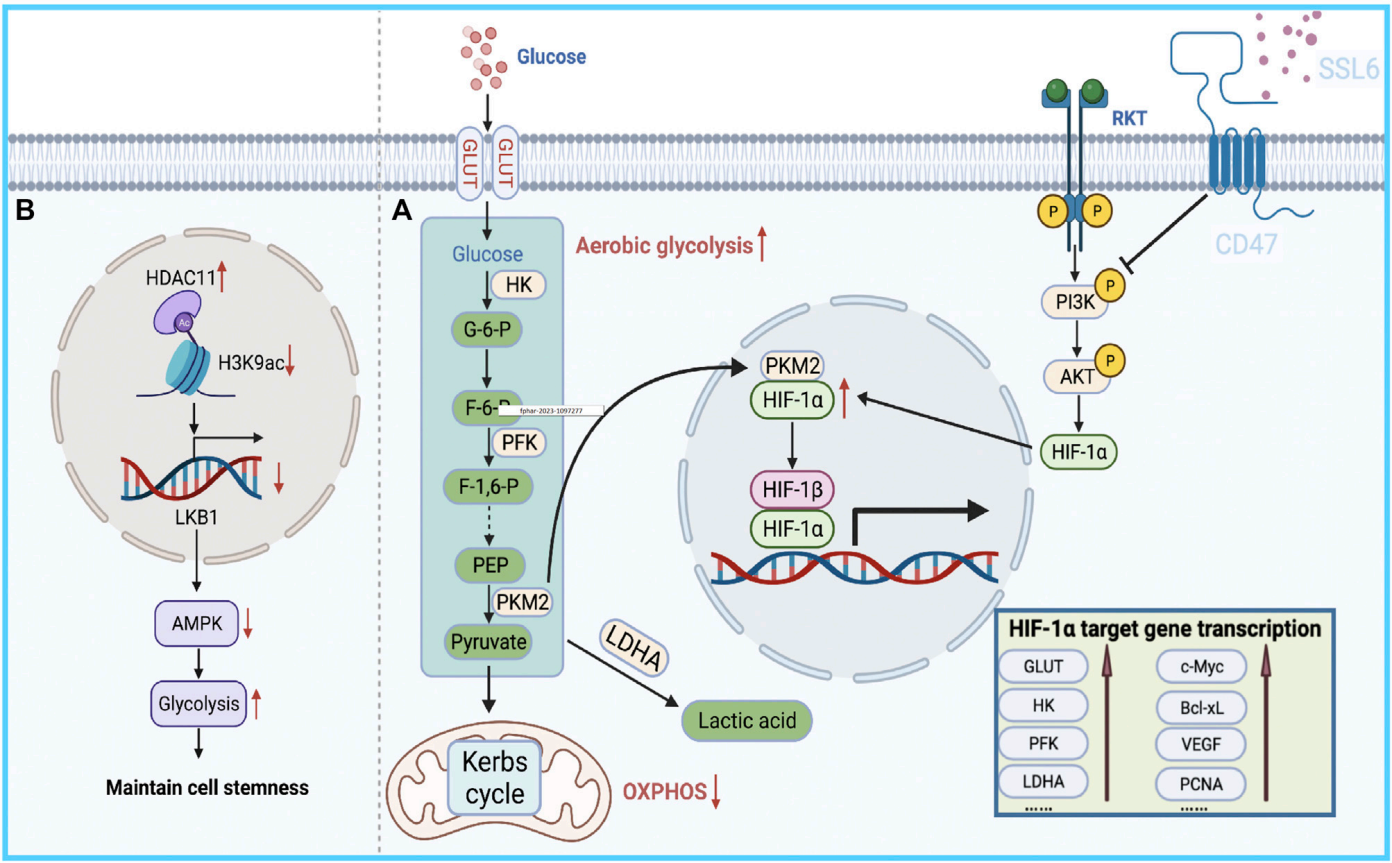
Bioenergetic propensity of HCC cells to utilize glycolysis is associated with sorafenib resistance. **(A)** HIFs mediate the primary transcriptional response to hypoxic stress and promote the expression of glycolysis-regulating enzymes. **(B)** HDAC11 inhibits the transcription of LKB1 by regulating histone acetylation in the promoter region of LKB1, thereby blocking AMPK signaling and inhibiting the glycolytic pathway, which in turn maintains tumor cell stemness.

This reprogrammed cancer metabolism is characterized by enhanced glycolysis and inhibition of oxidative phosphorylation (Chow et al., 2013; Du and Shim, 2016; Nieto et al., 2016; Gluck et al., 2019; Tan et al., 2019; Galle et al., 2020; Zhang et al., 2021a; Zhao et al., 2021a; Vishnoi et al., 2022), known as the Warburg effect (Stine et al., 2022). It has been reported that the bioenergetic propensity to utilize glycolysis is closely related to sorafenib resistance; thus, inhibiting glycolysis and activating oxidative phosphorylation can overcome intrinsic and acquired sorafenib resistance in HCC cells (Shen et al., 2013). Rate-limiting enzymes in glycolysis, such as 6-phosphofructose-1-kinase, pyruvate kinase, and hexokinase, are activated in sorafenib-resistant HCC cells. Consequently, inhibiting these enzymes to overcome sorafenib resistance is considered an effective treatment strategy (Li et al., 2017; Feng et al., 2019). Further exploration of the relationship between glycolysis and sorafenib resistance revealed that HIF-1a plays an important role in regulating glycolysis and apoptosis. HIFs mediate the primary transcriptional response under hypoxic stress and enhance the expression of several genes involved in glycolysis (Semenza, 2013). Therefore, HIF-1a inhibition might represent a strategy to overcome sorafenib resistance. Meanwhile, the PI3K/Akt pathway is closely related to glucose metabolism in tumor cells, and it is also involved in the regulation of HIF-1 α expression, indicating that the PI3K/Akt/HIF-1 α pathway plays a key role in the synergistic effect of hypoxia and the Warburg effect. Zhang et al. found that microbial-derived staphylococcal superantigen-like protein 6 could inhibited glycolysis by blocking the activation of PI3K/Akt/HIF-1 by CD47 to enhance the sensitivity of HCC cells to sorafenib (Zhang et al., 2020) (Figure 2A). In addition, glycolysis is closely related to CSCs. In HCC, compared with the effects of non-CSCs, CSCs exhibited a higher rate of glycolysis and higher expression of glycolytic genes; thus, inhibiting glycolysis could reduce the number of CSCs to overcome sorafenib resistance (Schieber and Chandel, 2013; Shen et al., 2015). Bi et al. found that loss of the histone deacetylase HDAC11 increased the transcription of LKB1, a serine/threonine kinase, by promoting histone acetylation in the LKB1 promoter region, which activated the AMPK signaling pathway and inhibited the glycolytic pathway, resulting in the inhibition of tumor cell stemness and the improvement of sorafenib resistance (Bi et al., 2021) (Figure 2B). Therefore, the resistance profile of HCC cells to sorafenib can be effectively improved by regulating the glycolytic level of tumor cells.

## Autophagy

Autophagy is the main intracellular degradation system that eliminates damaged intracellular organelles and misfolded proteins (Zhao et al., 2021b). It has been documented that autophagy has a paradoxical relationship in the development of resistance to sorafenib treatment in HCC (Zhong et al., 2016; Yazdani et al., 2019). On the one hand, researchers found that sorafenib protected tumor cells by activating autophagy in parental HCC cells. Some researchers found that sorafenib resistance associated with CD24 (CSC marker) is accompanied by the activation of autophagy, and resistance can be blocked by inhibiting autophagy using pharmacological inhibitors or knocking out autophagy-related genes. In further studies, investigated revealed that CD24 overexpression leads to increased PP2A protein production and induces inactivation of the mTOR/AKT pathway, thereby increasing autophagy levels. These experimental results indicate that CD24 can lead to sorafenib resistance progression by activating autophagy in hepatoma cells (Lu et al., 2018). Another experiment also confirmed that in HCC, ANXA3-mediated autophagy activation and attenuation of the PKCδ/p38-dependent apoptotic signaling pathway are involved in the development of sorafenib resistance, and HCC cells can be resensitized to sorafenib by inhibiting the expression of ANXA3 protein (Tong et al., 2018).

On the other hand, in resistant cell lines, the protective effect of autophagy could be switched to a role in promoting cell death. Because continuous drug exposure can induce unbalanced apoptotic pathways, it leads to cell resistance to apoptosis (Neophytou et al., 2021). Autophagy, as an adaptive response, switches from cytoprotective activity to pro-death functioning when apoptotic signals decay (Su et al., 2013; Zhai et al., 2014; Booth et al., 2020; Chen et al., 2021). Therefore, researchers found that inhibition of autophagy further reduced sorafenib sensitivity in sorafenib-resistant HCC cells, and conversely, inhibition of Akt induced a switch of autophagy from cytoprotective to pro-death mechanisms, thereby reversing acquired resistance to sorafenib (Neophytou et al., 2021). Because of the dual role of autophagy in the process of sorafenib resistance, additional caution is needed for clinical drugs that induce autophagy.

## Non-coding RNAs

**FIGURE 3:**
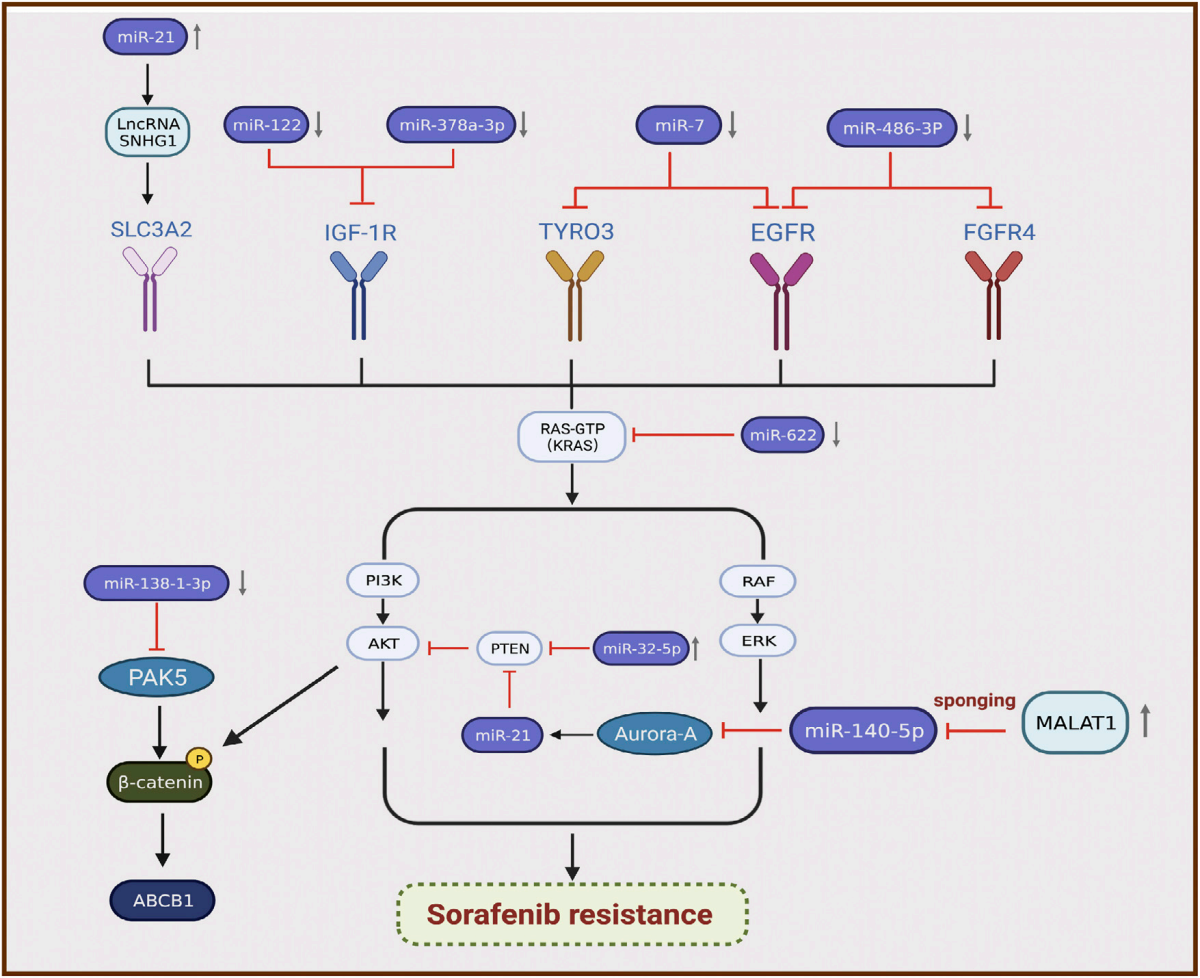
Differential expression of miRNAs is associated with the development of sorafenib resistance in hepatocellular carcinoma.

MicroRNAs (miRNAs) are sequences with an average length of 22 nucleotides that regulate target mRNA expression by binding to a short complementary sequence in the 3 ʹ -UTR region of mRNAs (Sun and Lai, 2013). In mammalian cells, miRNAs complex with Argonaute and Dicer to form RNA-induced silencing complex and guide them to cleave complementary mRNAs to achieve gene silencing, thereby inhibiting protein synthesis (Meister and Tuschl, 2004). Recent studies illustrated that the differential expression of miRNAs is closely related to sorafenib resistance in HCC, and most miRNAs exhibit lower expression in resistant tumor tissues than in normal tissues (Wei et al., 2019). For instance, miR-622 inhibits the expression of KRAS, leading to inhibition of the RAF/MAPK and PI3K/AKT pathways, suppression of HCC growth, and enhancement of sorafenib sensitivity (Dietrich et al., 2018). Therefore, miR-622 expression can be used as an auxiliary diagnostic tool to predict the response to sorafenib treatment in patients with HCC. Kabir et al. found that miR-7 is a potent tumor suppressor in human HCC, and TYRO3 is a novel functional target of miR-7 (Kabir et al., 2018). TYRO3 is a member of the TAM family of RTKs, and aberrant expression of the TYRO3/PI3K/AKT signaling pathway is a novel mechanism of acquired resistance to sorafenib in HCC. Experimental data illustrated that miR-7 overexpression could effectively silence TYRO3 expression in sorafenib-sensitive and sorafenib-resistant Huh-7 cells, thereby overcoming sorafenib Table 1 resistance in HCC caused by abnormal TYRO3 expression (Kabir et al., 2018). Ji et al. found that miR-486-3p was significantly downregulated in sorafenib-resistant HCC cell lines, further validating FGFR4 and EGFR as targets of miR-486-3p, and overexpression of miR-486-3p in combination with sorafenib could significantly inhibit tumor growth in a sorafenib resistance model (Ji et al., 2020). Li et al. detected significant downregulation of miR-138-1-3p and upregulation of PAK5 in sorafenib-resistant HCC cell lines. They found that PAK5 elevated the phosphorylation and nuclear translocation of β-catenin, thereby increasing the transcriptional activity of the multidrug resistance protein ABCB1, indicating that miR-138-1-3p mediates sorafenib resistance by negatively regulating PAK5 (Li et al., 2021). IGF-1 receptor (IGF-1R) is a major member of the tyrosine protein kinase receptor family that plays an important role in maintaining the malignant phenotype and anti-apoptosis of tumors. Overexpression of IGF-1R and its ligand IGF-1 is associated with tumor progression. Studies illustrated that IGF signaling is enriched in tumors with acquired resistance to sorafenib (Kouyos et al., 2014). Recently, two groups of researchers demonstrated that miR-122 and miR-378a-3p negatively regulated IGF-1R expression. Xu et al. found that IGF-1R could be activated by the ectopic downregulation of miR-122 to counteract sorafenib-induced apoptosis, thereby inducing sorafenib resistance (Xu et al., 2016). Similarly, Lin et al. confirmed that decreased XPO5 expression prevented the maturation of miR-378a-3p, which resulted in overexpression of IGF-1R and counteracted the effect of sorafenib-induced apoptosis (Lin et al., 2020). Mechanistically, downregulation of IGF-1R by miR-122 and miR-378a-3p contributes to activation of the RAS/RAF/ERK signaling pathway, which is associated with drug resistance (Xu et al., 2016; Lin et al., 2020) (Figure 3).

**TABLE 1:** Summary of previous studies with the mechanisms of receptor tyrosine kinase drug resistance in HCC.

Drug	Type of drug resistance	Mechanism of drug resistance	Reasons responsible	References
Sorafenib	Primary drug resistance	Mutation of EGFR	Dysregulation of EGFR and HER-3	Hsieh et al. (2011)
		Enrichment of CSC	LSD1 and activation of β-catenin	Lei et al. (2015)
			EPHB2/TCF1/EPHB2/β-catenin	Leung et al. (2021)
	Acquired drug resistance	Compensatory activation of the PI3K/Akt pathway	Activation of Akt	Chen et al. (2011)
		Compensatory activation of the MAPK/ER K pathway	Production of HGF and phosphorylation of c-Met	Han et al. (2017)
		EMT	Ets- 1-GPX2	Gluck et al. (2019)
			TNF-α/NF-κB/EM	Tan et al. (2019)
		Metabolic reprogramming	Activation of Rate	Li et al. (2017)
			limiting enzyme PI3K/Akt/HIF- 1α	Zhang et al. (2020)
			HDAC11/LKB1	Bi et al. (2021)
		Autophagy	The protective effect of autophagy	Lu et al. (2018), Lin et al. (2020), Tong et al. (2018)
			The pro-death mechanism of autophagy	Neophytou et al. (2021)
		Non-codingRNAs	MicroRNAs and LncRNAs	Table 2
		Evasion of apoptosis	Deficiency of PUMA	Dudgeon et al. (2012)
			Highly expression of FGFR4	Repana and Ross (2015)
		Dysregulation of cell cycle control	E2F1-Rb-cyclin E1	Hsu et al. (2016)
Lenvatinib	Primary Drug resistance	Activation of FGFR1/FGFR/VEGFR	High levels of FGFR1	Yamauchi et al. (2020)
		Enrichment of CSC	CD73-SOX9	Ma et al. (2020)
	Acquired drug resistance	High levels of EGFR	EGFR/PAK2/ERK5	Jin et al. (2021)
		Loss of NF1 and DUSP9	PI3K/AKT and MAPK/ERK	Lu et al. (2021)
		Non-coding RNAs	LncRNA MT1JP	Yu et al. (2021)
			LncRNA XIST	Duan et al. (2022)
			circMED27	Zhang P et al. (2021)
Regorafenib	Acquired drug resistance	EMT	Pin1/Gli1/Snail/E-cadherin	Wang et al. (2019)
		Sphk2	NF-κB and activation of STAT3	Shi et al. (2020)
		Activation of TGF-β signaling	Wnt/β-catenin	Karabicici et al. (2021)
		TOP2A	Wnt/β-catenin	Wang et al. (2022)
Cabozantinib	Primary drug resistance	Low levels of c-Met	C-Met	Gao et al. (2021)

By contrast, certain miRNAs are overexpressed in HCC. Fu et al. revealed that miR-32-5p was significantly upregulated in multidrug-resistant cell lines and positively correlated with low PTEN expression as well as poor prognosis (Fu et al., 2018). Overexpression of miR-32-5p activates the PI3K/Akt pathway by inhibiting PTEN and further induces multidrug resistance by regulating angiogenesis and EMT (Fu et al., 2018). Li et al. found that sorafenib induced the translocation of miR-21 to the nucleus and promoted the expression of the lncRNA small nucleolar RNA host gene 1, resulting in upregulation of SLC3A2 and activation of the Akt pathway, which is involved in the progression of sorafenib resistance (Li et al., 2019). MiRNAs can also interact with lncRNAs to participate in the progression of sorafenib resistance. Fan et al. verified that the lncRNA MALAT1 regulates Aurora-A expression through the sponge miR-140-5p, which promotes sorafenib resistance in HCC cells (Fan et al., 2020) (Figure 3). MALAT1 therefore has the potential to serve as a novel target for prognostic prediction and therapeutic strategies in patients with HCC treated with sorafenib.

Several miRNAs are widely involved in the development of sorafenib resistance in patients with HCC by regulating the differential expression of sorafenib-targeted kinases. Recent studies demonstrated that miRNAs can be used as tissue-specific biomarkers to predict sorafenib resistance in patients with HCC, and the sensitivity of resistant cells to sorafenib can be effectively improved by artificially altering the content of miRNAs within HCC cells. Simultaneously, the involvement of miRNAs in regulating sorafenib resistance in HCC is a multi-level and multi-target process, and thus, the synergistic regulation of multiple miRNAs can be considered when studying the expression of miRNAs within HCC cells. In studying strategy to reverse sorafenib resistance, miRNAs represent an important link that must be considered, and studies targeting miRNAs will yield great benefits for the prognosis of patients with sorafenib-resistant HCC.

## Apoptosis resistance and deregulated cell cycle control

Evasion of apoptosis is a common feature of cancer cells that is tightly associated with drug resistance (Shahar and Larisch, 2020). Cancer cells overexpress many proteins that play important roles in resisting activation of the apoptotic cascade, such as Bcl-2, Bcl-xL, and Mcl-1 (Mohammad et al., 2015). Dudgeon et al. found that sorafenib was able to kill cancer cells by activating PUMA (an apoptotic modulator upregulated by p53) (Dudgeon et al., 2012). As a large subclass of the Bcl-2 protein family, PUMA, a BH3-domain only protein, is a key initiator of apoptosis in cancer cells (Edwards et al., 2013). PUMA deficiency abolished apoptosis and caspase activation induced by sorafenib, whereas BH3 analogs enhanced the anti-cancer effect of sorafenib and restored the sensitivity of resistant cells to sorafenib (Dudgeon et al., 2012). As an oncogenic driver in HCC, FGF19, with its main receptor FGFR4, is highly expressed in primary HCC, and its new role in sorafenib resistance was reported (Repana and Ross, 2015). By overexpressing FGF19, tumor cells inhibit ROS generation and apoptosis induced by sorafenib. Importantly, targeting the FGF19/FGFR4 axis by administering ponatinib, a third-generation Table 2 inhibitor for chronic myelogenous leukemia treatment, can overcome resistance to sorafenib in HCC by enhancing ROS-related apoptosis (Gao et al., 2017). These findings suggest that exploring apoptotic mechanisms provides a theoretical basis for improving cell sensitivity to targeted therapy.

**TABLE 2:** Previous studies that show the involvement of miRNAs in sorafenib resistance in HCC.

Name	Effects on sorafenib resistance	Target	Reference
miR-622	Inhibiting	KRAS	Dietrich et al. (2018)
miR-7	Inhibiting	TYRO3	Kabir et al. (2018)
miR-486-3p	Inhibiting	FGFR4/EGFR	Ji et al. (2020)
miR-138-1-3p	Inhibiting	PAK5	Li et al. (2021)
miR-122	Inhibiting	IGF-1R	Xu et al. (2016)
miR-378a-3p	Inhibiting	IGF-1R	Lin et al. (2020)
miR-32-5p	Promoting	PTEN	Fu et al. (2018)
miR-21	Promoting	LncRNA SNHG1	Li et al. (2019)
miR-140-5p	Inhibiting	lncRNA MALAT1	Fan et al. (2020)

The following references were added.

Furthermore, dysregulation of cell cycle control is a hallmark of cancer, and overexpression of cyclins is usually closely related to tumorigenesis progression (Suski et al., 2021). Many studies demonstrated that synergistic use of cell cycle inhibitors enhanced sorafenib anticancer activity as well as partially antagonize multidrug resistance (Reiter et al., 2019; Lee et al., 2020; Yu et al., 2020). Hsu et al. found that the regulation of the E2F1–Rb–cyclin E1 complex might play a crucial role in mediating sorafenib resistance in HCC cells, and depletion of cyclin E1 expression reversed sorafenib resistance in HCC cells in terms of cell growth and apoptosis induction (Hsu et al., 2016). In addition, the combination of sorafenib and CDK inhibitors might improve the efficacy of sorafenib in the treatment of HCC (Hsu et al., 2016).

## Lenvatinib resistance

Lenvatinib is an oral inhibitor of multiple RTKs including VEGFR1–3, FGFR1–4, platelet PDGFR α, RET, and KIT (Matsui et al., 2008). It became the second approved first-line treatment for patients with advanced HCC in 2018, and its efficacy against some RTKs was superior to that of sorafenib (Kudo et al., 2018; Matsuki et al., 2018). However, similar to sorafenib, lenvatinib initially controls tumors well, but resistance gradually develops over time.

In contrast to classical cytotoxic agents, lenvatinib targets specific molecular and cancer signaling pathways. Thus, diversity of genetic drivers is decisive for primary resistance to lenvatinib compared to sorafenib. Kinases such as FGFR1, FGFR4, and VEGFR, as targets of lenvatinib, have been shown to influence lenvatinib efficacy at high and low levels of expression (Zhao et al., 2021c; Zhao et al., 2021d; Shi et al., 2021). FGFR1 levels were shown to be an independent predictor of response to rosuvastatin (Yamauchi et al., 2020). Certain alkaloid extracts such as Oxysophocarpine and Sophoridine can improve lenvatinib sensitivity by inhibiting these target kinases (Zhao et al., 2021c; Zhao et al., 2021d; Shi et al., 2021). In hepatocellular carcinoma, CSC initiates tumor development, induces tumor development and regulates chemoresistance (Lee et al., 2022). Ma et al. found CD73 to be a potential marker for CSC recognition in HCC, overexpression of CD73 rendered HCC cells significantly resistant to rosuvastatin, and purified CD73^+^cells showed excellent resistance compared with CD73−cells (Ma et al., 2020). Mechanistically, CD73 maintains CSC traits by upregulating SOX9 expression and maintaining the stability of its protein, which would be a potential target to overcome resistance to Lenvatinib (Ma et al., 2020).

Similarly, patients with HCC developed varying degrees of Acquired resistance to lenvatinib, and the development of this resistance involved alterations in multiple intracellular signaling pathways. JIN et al. found that high levels of EGFR conferred resistance to lenvatinib in HCC patients (Jin et al., 2021). This is due to inhibition of FGFR by lenvatinib treatment resulting in aberrant activation of the EGFR/PAK2/ERK5 signaling axis (Jin et al., 2021). Lenvatinib inhibits its downstream pathway by inhibiting kinases, in which aberrant activation of PI3K/Akt and MEK/ERK signaling pathways alters drug resistance in HCC cells. Lu et al. performed a genome-wide screen of HCC cells treated with or without Lenvatinib and identified NF1 and DUSP9 as key factors for Lenvatinib resistance (Lu et al., 2021). Loss of NF1 reactivates PI3K/AKT as well as MAPK/ERK pathways in HCC cells inhibited by Lenvatinib, loss of DUSP9 activates MAPK/ERK pathway and induces phosphorylation of AKT and ERK to induce Lenvatinib resistance (Lu et al., 2021). Levels of autophagy also influence sustained therapeutic efficacy of lenvatinib. Lu et al. found that LAPTM5 could promote intrinsic macroautophagic/autophagic flux by facilitating autolysosome formation to drive lenvatinib resistance (Pan et al., 2022). Non-coding RNAs are also involved in the development of lenvatinib resistance, and Yu et al. confirmed that the lncRNA MT1JP was upregulated to inhibit apoptotic signaling pathways in LR-HCC cells, thereby reducing the sensitivity of hepatocytes to lenvatinib (Yu et al., 2021). Recently, Anqi Duan et al. first reported that long non-coding RNA XIST can promote lenvatinib resistance in hepatocellular carcinoma cells through epigenetic inhibition of NOD2 (Nucleotide-binding oligomerization domain 2) (Duan et al., 2022). Mechanistically, lncXIST is able to bind to the histone modifying enzyme EZH2, which acts as a core subunit of the PRC2 complex (Polycomb Repressive Complex 2) and promotes transcriptional silencing by catalyzing trimethylation of histone H3K27, thus negatively regulating NOD2 expression (Liu et al., 2021; Bae et al., 2022). EZH2 has been reported to be overexpressed in HCC and associated with poor prognosis ^[ 106]^. The results of this study further suggest EZH2 as a target to overcome lenvatinib resistance in HCC cells. Furthermore, Zhang et al. found that the circRNA circMED27 was significantly upregulated in HCC serum, and it acts as competitive endogenous RNA for miR-655-3p (Duffy and Greten, 2017). It act as a sponge to adsorb miR-655-3p and then upregulate the expression of ubiquitin-specific peptidase 28 (USP28), promoting the resistance of HCC cells to lenvatinib (Zhang et al., 2021b). Research into the resistance mechanism of lenvatinib, as the second first-line HCC treatment, will benefit the treatment of patients with HCC.

## Resistance to other kinase inhibitors

After many patients failed treatment with sorafenib because of multi-mechanism resistance, regorafenib was approved by the FDA in 2017 for the second-line treatment of patients with unresectable HCC (Bruix et al., 2017; Duffy and Greten, 2017). Therefore, current studies on regorafenib resistance have focused more on the mechanism of its Acquired resistance. Pin1, a unique phosphorylation-specific peptidyl-prolyl cis-trans isomerase, is a common regulator of a variety of oncogenic signaling networks, and it was identified as a key isomerase in regulating HCC progression (Wei et al., 2015; Pu et al., 2018). Wang et al. demonstrated that inhibition of Pin1 can reverse acquired resistance to regorafenib in HCC in part by inhibiting EMT through the Gli1/Snail/E-cadherin pathway (Zhang et al., 2021b). Their study revealed for the first time the molecular mechanism of regorafenib resistance in HCC, suggesting that Pin1 inhibitors will be an alternative treatment class for the treatment of aggressive and regorafenib-resistant HCC (Wang et al., 2019). Recently, many findings illustrated that overexpression of sphingosine kinase 2 (SphK2) is associated with drug resistance in tumor cells (Liu et al., 2016). Shi et al. first demonstrated that SphK2/S1P is a key regulator mediating regorafenib resistance to HCC through NF- κ B and STAT3 activation (Shi et al., 2020). Thus, ABC294640, a selective inhibitor of SphK2, exhibited high potential to increase the sensitivity of regorafenib-resistant HCC cells to the drug (Shi et al., 2020). Karabici et al. found that HCC tumors with abnormal Wnt/β-catenin activation may have higher intrinsic regorafenib resistance (Karabicici et al., 2021). After this, Zongwen Wang et al. found that silencing TOP2A, a key pro-oncogene in a variety of tumors, blocked the EMT process and reversed acquired resistance to regorafenib through the Wnt- β-catenin pathway (Wang et al., 2022). In addition, regorafenib resistant cells have enhanced TGF- β signaling activity and significantly higher *in vivo* migration ability, which could be reversed upon TGF β -R1 inhibition (Karabicici et al., 2021). Therefore, the combined use of TGF- β pathway inhibitors and regorafenib is a promising method for sensitization and prevention of tumor recurrence in patients with HCC with acquired regorafenib resistance. Regorafenib is structurally similar to sorafenib, but regorafenib is more potent against VEGFR kinases, and this potent anti-angiogenic effect provides a precondition for regorafenib to improve resistance to anti-PD-1/PD-L1 therapy (Liu et al., 2022). Recently, some preclinical findings suggest that regorafenib exhibits anti-immunosuppressive properties as an anti-angiogenic agent (Schmittnaegel and De Palma, 2017; Arai et al., 2019). For example, regorafenib can promote anti-tumor immunity by regulating macrophages and increasing the proliferation and activation of CD8 +T cells, so the combination of regorafenib with immune checkpoint inhibitors can be regarded as a new dosing strategy, which may delay the development of its resistance (Granito et al., 2021).

Cabozantinib is a tyrosine kinase inhibitor with potent activity against MET, VEGFR2, RET, KIT, AXL, and FLT3. These kinases have been implicated in HCC progression and the development of resistance to sorafenib (Yakes et al., 2011). Cabozantinib was approved in 2019 for patients with advanced HCC who have been treated with sorafenib (Abou-Alfa et al., 2018). c-MET plays a key role in the occurrence and development of HCC, which is related to HCC cell proliferation, survival, and invasiveness, angiogenesis and the development of resistance to chemotherapeutic drugs (Venepalli and Goff, 2013; Bouattour et al., 2018). c-MET is the main target of the anti-tumor activity of cabozantinib. However, Gao et al. found that HCC cells with low c-Met levels exhibited primary resistance to c-MET inhibitors, and the combination of cabozantinib and the mTOR inhibitor rapamycin exerted synergistic inhibitory effects on cell proliferation and tumor growth in resistant cells (Gao et al., 2021; Shang et al., 2021). Therefore, these results suggest that the development of cabozantinib resistance can be partially avoided using rational combinations. Increasing evidence supports the immunostimulatory effects of cabozantinib, and the potential interaction between cabozantinib and anti-PD-1 inhibitors has now been investigated in preclinical studies (Cammarota et al., 2022). In the COSMIC-021 trial (NCT03170960), the combination of cabozantinib plus atezolizumab showed encouraging activity in a variety of solid tumors (Cammarota et al., 2022). Cabozantinib plus afatinib may be a new first-line treatment option for patients with advanced hepatocellular carcinoma, and such a combination may slow cabozantinib resistance (Kelley et al., 2020).

Donafenib, a deuterium derivative of sorafenib. By inhibiting phosphorylation of serine/threonine kinases and by blocking RTK signaling, donafenib shows similar antitumor activity as sorafenib for the advanced HCC patients (Keam and Duggan, 2021). On July 9th of 2021, according to Chinese NMPA, donafenib produced by Suzhou Zelgan was approved as a treatment for unresectable HCC patients without systemic therapy (Keam and Duggan, 2021). Because donafinil shares similar targeting sites as well as utility with sorafenib, we speculate that it may have a potential resistance mechanism similar to sorafenib. However, there is no evidence to prove the occurrence of drug resistance, which needs further study and exploration.

## Possible strategies

Systemic therapy plays an important role in the treatment of liver cancer. On the one hand, patients with advanced HCC miss the opportunity for surgical resection or local treatment. On the other hand, the majority of patients with HCC have to receive systemic treatment after resection treatment because of the high recurrence rate of HCC (Villanueva, 2019). It is worth mentioning that before sorafenib was approved as the first-line treatment for advanced HCC in 2008, there were no curative treatments for patients with advanced HCC.

Although receptor tyrosine kinase targeted drugs such as sorafenib prolong the survival of patients with advanced HCC, the occurrence of drug resistance greatly reduces their clinical benefits. It is gratifying that with the development of molecular biology, the mechanism of kinase drug resistance has gradually been revealed, thereby providing key insights into clinical treatment (Kannaiyan and Mahadevan, 2018). First, in the case of sorafenib, because of the genetic heterogeneity of tumors, many patients exhibit primary resistance characteristics during the initial treatment. Thus, it is possible to predict the efficacy of sorafenib by defining molecular markers related to resistance through tumor genome sequencing technology in clinical practice. These novel molecular markers might include EGFR and its downstream molecules or cellular markers affecting tumor stemness. Second, patients who develop acquired resistance after sorafenib treatment can be treated by adjusting the dose or changing to another targeted drug. For example, immune checkpoint inhibitors can be used as alternative treatment options. The combination of atezolizumab and the antiangiogenic agent bevacizumab prolonged OS *versus* sorafenib monotherapy in patients with advanced HCC (Wang et al., 2014). In addition, drug intervention against some specific targets can also be used to resensitize tumors to sorafenib. The Akt inhibitor GDC0068 can reverse acquired resistance to sorafenib by switching autophagy from cytoprotective to pro-death activity (Zhai et al., 2014). TNF-α/NF-κB/EMT signaling inhibition using ulinastatin overcomes sorafenib resistance in HCC (Tan et al., 2019).

Despite significant clinical benefit of lenvatinib as a VEGFR inhibitor, dose reduction or discontinuation is generally required due to its severe toxicity (Nakazawa et al., 2015). In addition, almost all cancers can show resistance to VEGFR inhibitors through various mechanisms (Nakazawa et al., 2015). Clinical studies have shown that serum angiopoietin-2 (Ang2) levels are considered as potential biomarkers of VEGFR inhibitor response in several cancers (Miyahara et al., 2011; van der Veldt et al., 2012). Golvatinib is an inhibitor of c-Met and Tie2 (Wang et al., 2012). Preclinical studies have shown that combining lenvatinib with golvatinib can sensitize tumors to lenvatinib and may reduce the clinical dose of lenvatinib (Nakazawa et al., 2015). In addition, there was one experiment show that inhibition of epidermal growth factor receptor (EGFR) is synthetic lethal with lenvatinib in liver cancer (Vakil and Trappe, 2022). Therefore, lenvatinib in combination with EGFR inhibitors (gefitinib, etc.) is effective in improving lenvatinib resistance in patients with a high EGFR profile and is a promising combination strategy (Vakil and Trappe, 2022).

The molecular pathogenesis of HCC is very complex and involves different pathways and molecular aberrations such as RAS/RAF/MEK/ERK, PI3K/AKT/mTOR, VEGF, c-Met, and HDACs, simultaneous or sequential elimination of the function of these key pathways or key molecules may improve the therapeutic dilemma of HCC patients. Inhibition of multiple nodes of a pathway, either downstream or upstream of a driver oncogene, through dual blockade of oncogenic signaling has been shown to be a reasonably effective way to prolong the response to oncogenic pathway inhibition. On the other hand, tumor survival can be curbed by using two or more drugs in the same route or multiple drugs that simultaneously target two parallel routes (Jin et al., 2022). Currently, in the case of sorafenib, it has been combined with anti-angiogenic agents, MEK/ERK pathway inhibitors, mTOR pathway inhibitors, histone deacetylase inhibitors, EGF/EGFR pathway inhibitors, and HGF/c-Met pathway inhibitors, but to date, treatment involving sorafenib-containing combination therapy has not been successful in phase III trials (Huang et al., 2020). Among them, drug toxicity amplification in combination therapy trials has become a bottleneck in currently translating positive preclinical experiments into HCC clinical trials. Therefore, it is recommended that drug combinations with no or less overlapping toxicity profiles and drug interactions minimize the risk of amplification of adverse reactions (Gao et al., 2015).

Receptor tyrosine kinase inhibitors in the treatment of HCC currently face barriers to resistance to mutations in genes encoding receptors and effector factors. For example, changes in kinase gating residues can hinder inhibitor binding by altering hydrophobic interactions, as suggested by the Thr 315 (encoded by ACT) mutation in BCR-ABL kinase, which leads to imatinib resistance (Mou et al., 2021). Elevated tissue expression of pERK and VEGFR-2 predicts adverse outcomes in advanced HCC treated with sorafenib (Personeni et al., 2013; Negri et al., 2015). SNPs in the VEGFR2 gene are significantly associated with clinical outcomes in HCC patients (Hack et al., 2020). Understanding and exploring the mechanism of resistance mutations including EGFR and other receptor kinase domain and optimizing future coping strategies are urgent problems to be solved in HCC kinase inhibitor therapy. Also, given the spatiotemporal heterogeneity of tumors as well as individual differences in resistance mechanisms, tissue biopsies and genetic testing are recommended for patients who still experience disease progression following TKI therapy. This helps to identify gene kinase domain mutations, clarify the resistance mechanism of TKIs, carry out more targeted basic and clinical translational research, and establish more accurate and effective treatment strategies.

Traditional cancer treatments are based on the continuous administration of fixed doses of single or multiple drugs, using the MTD (maximum tolerated dose) to kill as many cancer cells as possible to obtain the greatest therapeutic effect. However, increasing evidence suggests that treatments aimed at eliminating susceptible subpopulations result in altered tumor microenvironment favoring resistant subpopulations, which enhances the probability and rate of resistance emergence (Chatterjee and Bivona, 2019). Increasing research has focused on dose strategies to combat resistance in tumor progression in combination therapy, and both extremes of too high and too low doses may unnecessarily accelerate the spread of resistance (Vakil and Trappe, 2021; Kouyos et al., 2014). Some results suggest that maintaining a low-dose treatment strategy is advantageous when the size of the patient‘s tumor is tolerable, as it allows the susceptible clonal population to survive and compete with the resistant subpopulation to prevent the resistant population from propagating uncontrollably and taking over the entire tumor, thus having more feared consequences for the patient (Morgillo et al., 2007; Vakil and Trappe, 2021). In addition, in situations where the patient‘s immune response increases over time, delaying the emergence of resistance may provide sufficient time for immunity to help prevent resistance (Hsieh et al., 2011; Ezzoukhry et al., 2012; Hagiwara et al., 2012; Yarden and Pines, 2012; Vidal et al., 2014; Gao et al., 2021b). These interesting findings suggest whether the administered dose of TKIs can be minimized to delay the development of drug resistance phenomenon under the premise of maintaining the survival status of patients.

In conclusion, in this paper, we reviewed the resistance mechanisms of small-molecule kinase inhibitors in the treatment of HCC and corresponding improved strategies, hoping to improve the outcomes of patients with HCC.
