# Erratum: Drug resistance mechanism of kinase inhibitors in the treatment of hepatocellular carcinoma

**DOI:** 10.3389/fphar.2023.1188062

**Published:** 2023-04-03

**Authors:** Frontiers Production Office

**Affiliations:** Frontiers Media SA, Lausanne, Switzerland

**Keywords:** hepatocellular carcinoma, drug resistance, sorafenib, lenvatinib, regorafenib, cabozantinib

Due to an error in the editorial process, an incorrect version of the article was published. Significant textual revisions to the published article are detailed below.

The previous version is available in the Supplementary Material of this Erratum. The article has now been updated with the correct version. The publisher apologizes for this error.

A correction has been made to the section “Primary Drug Resistance”, subsection “Epidermal growth factor receptor (EGFR)”. This section has been removed and its contents merged with the section entitled “Tumor heterogeneity and EGFR”.

A correction has been made to the section “Acquired Drug Resistance”, subsection “PI3K/AKT and MAPK/ERK signaling pathways”. This section has been removed and replaced with the section entitled “EGFR and HGF/cMet mediated signaling pathway”.

A spelling mistake was corrected in the keywords section of this article. “Carcinom” was corrected to “carcinoma”.

Additional references have been added to the published article. Details of these references can be found in the "References" section of this article.

Tables 1, 2 have been added to the published article.

**TABLE 1:** Summary of previous studies with the mechanisms of receptor tyrosine kinase drug resistance in HCC.

Drug	Type of drug resistance	Mechanism of drug resistance	Reasons responsible	References
sorafenib	Primary drug resistance	Mutation of EGFR	Dysregulation of EGFR and HER-3	Hsieh et al. (2111)
		Enrichment of CSC	LSD1 and activation of β-catenin	Lei et al. (2015)
			EPHB2/TCF1/EPHB2/β-catenin	Leung et al. (2021)
	Acquired drug resistance	compensatory activation of the PI3K/Akt pathway	Activation of Akt	Chen et al. (2011)
		compensatory activation of the MAPK/ER K pathway	Production of HGF and phosphorylation of c-Met	Han et al. (2017)
		EMT	Ets- 1-GPX2	Gluck et al. (2019)
			TNF-α/NF-κB/EMT	Tan et al. (2019)
		Metabolic reprogramming	Activation of Rate limiting enzyme	Li et al. (2017)
			PI3K/Akt/HIF- 1α	Zhang et al. (2020)
			HDAC11/LKB1	Bi et al. (2021)
		Autophagy	The protective effect of autophagy	Lu et al. (2018), Tong et al. (2018), Lin et al. (2020b)
			The pro-death mechanism of autophagy	Neophytou et al. (2021)
		Non-coding RNAs	MicroRNAs and LncRNAs	Table 2
		Evasion of apoptosis	Deficiency of PUMA	Dudgeon et al. (2012)
			Highly expression of FGFR4	Repana and Ross (2015)
		Dysregulation of cell cycle control	E2F1-Rb-cyclin E1	Hsu et al. (2016)
Lenvatinib	Primary drug resistance	Activation of FGFR1/FGFR/VEGFR	High levels of FGFR1	Yamauchi et al. (2020)
		Enrichment of CSC	CD73-SOX9	Ma et al. (2020)
	Acquired drug resistance	High levels of EGFR	EGFR/PAK2/ERK5	Jin et al. (2021)
		Loss of NF1 and DUSP9	PI3K/AKT and MAPK/ERK	Lu et al. (2021)
		Non-coding RNAs	LncRNA MT1JP	Yu et al. (2021)
			LncRNA XIST	Duan et al. (2022)
			circMED27	Zhang et al. (2021)
regorafenib	Acquired drug resistance	EMT	Pin1/Gli1/Snail/E-cadhe rin	Wang et al. (2019)
		SphK2	NF-κB and activation of STAT3	Shi et al. (2020)
		Activation of TGF-β signaling	Wnt/β-catenin	Karabicici et al. (2021)
		TOP2A	Wnt/β-catenin	Wang et al. (2022)
cabozantinib	Primary drug resistance	Low levels of c-Met	C-Met	Gao et al. (2021)

**TABLE 2:** Previous studies that show the involvement of miRNAs in sorafenib resistance in HCC.

Name	Effects on sorafenib resistance	Target	Reference
miR-622	Inhibiting	KRAS	Dietrich et al. (2018)
miR-7	Inhibiting	TYRO3	Kabir et al. (2018)
miR-486-3p	Inhibiting	FGFR4/EGFR	Ji et al. (2020)
miR-138-1-3p	Inhibiting	PAK5	Li et al. (2021)
miR-122	Inhibiting	IGF-1R	Xu et al. (2016)
miR-378a-3p	Inhibiting	IGF-1R	Lin et al. (2020a)
miR-32-5p	Promoting	PTEN	Fu et al. (2018)
miR-21	Promoting	LncRNA SNHG1	Li et al. (2019)
miR-140-5p	Inhibiting	lncRNA MALAT1	Fan et al. (2020)
