# Supplementary material for: Erratum: Drug resistance mechanism of kinase inhibitors in the treatment of hepatocellular carcinoma
Source: Front Pharmacol. 2023 Apr 3;14:1188062. doi: 10.3389/fphar.2023.1188062 (PMC10107049; doi:10.3389/fphar.2023.1188062)
Supplement: Supplementary file 1 [file RelatedArticle1.pdf]

frontiers | Frontiers in Pharmacology

TYPE Review
PUBLISHED 13 February 2023
DOI 10.3389/fphar.2023.1097277

Check for updates

OPEN ACCESS

EDITED BY
Jiyao Sheng,
Jilin University, China

REVIEWED BY
Raúl Gonzalez Ojeda,
University of Galway, Ireland
Deniz Cansen Kahraman,
Middle East Technical University, Turkey
Nurbubu Moldogazieva,
I.M. Sechenov First Moscow State Medical University, Russia
Zunqiang Xiao,
Zhejiang Chinese Medical University, China

*CORRESPONDENCE
Shengtao Yuan,
yuanst@cpu.edu.cn
Yanyan Liu,
bloom0611@163.com

†These authors share first authorship

SPECIALTY SECTION
This article was submitted to Pharmacology of Anti-Cancer Drugs, a section of the journal Frontiers in Pharmacology

RECEIVED 13 November 2022
ACCEPTED 01 February 2023
PUBLISHED 13 February 2023

CITATION
Jiang L, Li L, Liu Y, Lu L, Zhan M, Yuan S and Liu Y (2023), Drug resistance mechanism of kinase inhibitors in the treatment of hepatocellular carcinoma. *Front. Pharmacol.* 14:1097277. doi: 10.3389/fphar.2023.1097277

COPYRIGHT
© 2023 Jiang, Li, Liu, Lu, Zhan, Yuan and Liu. This is an open-access article distributed under the terms of the Creative Commons Attribution License (CC BY). The use, distribution or reproduction in other forums is permitted, provided the original author(s) and the copyright owner(s) are credited and that the original publication in this journal is cited, in accordance with accepted academic practice. No use, distribution or reproduction is permitted which does not comply with these terms.

Drug resistance mechanism of kinase inhibitors in the treatment of hepatocellular carcinoma

Lei Jiang[1†], Luan Li[2†], Yongzhuang Liu[3†], Ligong Lu[1], Meixiao Zhan[1], Shengtao Yuan[3*] and Yanyan Liu[1*]

[1]Guangdong Provincial Key Laboratory of Tumor Interventional Diagnosis and Treatment, Zhuhai People's Hospital (Zhuhai Hospital Affiliated With Jinan University), Zhuhai, Guangdong, China, [2]Jiangsu Cancer Hospital, Jiangsu Institute of Cancer Research, The Affiliated Cancer Hospital of Nanjing Medical University, Nanjing, Jiangsu, China, [3]Jiangsu Key Laboratory of Drug Screening, China Pharmaceutical University, Nanjing, Jiangsu, China

Hepatocellular carcinoma (HCC) is the most common form of primary liver cancer, and it usually occurs following chronic liver disease. Although some progress has been made in the treatment of HCC, the prognosis of patients with advanced HCC is not optimistic, mainly because of the inevitable development of drug resistance. Therefore, multi-target kinase inhibitors for the treatment of HCC, such as sorafenib, lenvatinib, cabozantinib, and regorafenib, produce small clinical benefits for patients with HCC. It is necessary to study the mechanism of kinase inhibitor resistance and explore possible solutions to overcome this resistance to improve clinical benefits. In this study, we reviewed the mechanisms of resistance to multi-target kinase inhibitors in HCC and discussed strategies that can be used to improve treatment outcomes.

KEYWORDS

hepatocellular carcinom, drug resistance, sorafenib, lenvatinib, regorafenib, cabozantinib

Introduction

Liver cancer is the second leading cause of cancer-related death worldwide, with approximately 850,000 new cases occurring annually, and hepatocellular carcinoma (HCC) accounts for approximately 90% of primary liver cancers. Hepatitis B and C virus infection, alcohol intake, ingestion of the fungal metabolite aflatoxin B1, and non-alcoholic steatohepatitis are potential risk factors for HCC (Llovet et al., 2021). Currently, patients with early HCC can be cured by radical hepatectomy, liver transplantation, and local ablation. Patients with intermediate HCC receive local therapy (e.g., chemoembolization), whereas patients with advanced HCC only benefit from systemic therapy (Llovet et al., 2018). With the in-depth study of targeted therapy for HCC, there is increasing evidence that multi-target combination therapy has a significant synergistic anti-tumor effect. Sorafenib, a multi-target kinase inhibitor with anti-angiogenic and anti-proliferative effects, prolongs the overall survival (OS) of patients with advanced HCC from 8 months to 11 months, and it was the only systemic therapeutic agent used to treat HCC between 2007 and 2016 (Llovet et al., 2008). In 2018, lenvatinib became the second first-line treatment approved for patients with advanced HCC (Kudo et al., 2018). Meanwhile, regorafenib was approved by the FDA in 2017 for second-line treatment in patients with unresectable HCC. In 2019, cabozantinib was used for second-line treatment in patients who had previously been treated with sorafenib (Bruix et al., 2017; Abou-Alfa et al., 2018).

Although systemic therapy for patients with advanced HCC can prolong the median survival time, it often leads to treatment failure because of the development of tumor cell resistance to small-molecule kinase inhibitors, which has become a major obstacle to the clinical treatment of patients with advanced HCC. There are primary or secondary factors in the development of drug resistance in tumor cells. Primary resistance occurs at the beginning of drug treatment; that is, genetic heterogeneity of tumor cells leads to insensitivity to therapeutic drugs. Conversely, secondary resistance describes resistance in which, after a period of clinical benefit, small-molecule kinase inhibitors gradually become ineffective during treatment. The most common cause of acquired resistance is the activation of signaling pathways that bypass drug targets to maintain survival and proliferation. In fact, because small-molecule kinase inhibitors generally target a variety of signaling pathways, resistance occurs once the related compensatory cascade signaling pathways are activated. At the same time, a large number of new targets have also been found to be associated with HCC resistance (Tang et al., 2020). Determining the resistance factors of small-molecule kinase inhibitors and exploring the regimens that can be used to overcome or delay drug resistance has certain guiding significance for clinical treatment. Therefore, in this review, we summarized the results of recent studies on the potential mechanisms leading to resistance to small-molecule kinase inhibitors and explored some strategies that could be used to improve treatment outcomes.

Drug resistance of sorafenib

In 2007, sorafenib, a small-molecule targeted drug, was approved for the treatment of advanced liver cancer. In the following decade, sorafenib remained the only first-line targeted therapy for advanced HCC (Llovet et al., 2008). As an oral small-molecule kinase inhibitor, it inhibits intracellular serine/threonine kinases (including the Raf/MEK/ERK signaling pathway) and receptor tyrosine kinases (RTKs; including vascular endothelial growth factor receptors [VEGFR-1], VEGFR-2, VEGFR-3, platelet-derived growth factor receptor [PDGFR]-β, c-KIT, FMS-like tyrosine kinase 3 [FLT-3], and rearranged during transfection [RET]), thereby inhibiting tumor growth and angiogenesis (Tang et al., 2020). Although patients with HCC who received sorafenib exhibited a significant increase in mean OS, only a small number of patients obtained a real and long-term benefit from this therapy (Keating, 2017). Thus, elucidating the mechanism of sorafenib resistance is important for prolonging the survival of patients with HCC.

Primary drug resistance

Epidermal growth factor receptor (EGFR)

EGFR is a 170-kDa transmembrane glycoprotein composed of an extracellular domain that recognizes and binds specific ligands as well as an intracellular domain that acts as a protein kinase. Activated EGFR stimulates the activation of several signal transduction pathways (Morgillo et al., 2007). The expression and activation of EGFR and its major dimerization partner HER-3 (ErbB-3) are frequently dysregulated in HCC (Hsieh et al., 2011). Blivet-Van Eggelpoël et al. (2012) provided evidence that dysregulation of the EGFR/HER-3 signaling pathway limits the efficacy of sorafenib in treatment-naïve or acquired-resistant HCC cells. At the same time, dysregulation of the Ras-Raf-Mek-ERK, PI3K-Akt-mTOR, PLC-γ1, signal transducer and activator of transcription, and Src pathways downstream of EGFR are involved in tumor cell proliferation and apoptosis, which are tightly associated with sorafenib resistance (Yarden and Pines, 2012). Therefore, anti-EGFR therapy might improve the therapeutic benefit of sorafenib by alleviating primary resistance (Shang et al., 2021). Investigators have used several different methods to block the expression of EGFR, the kinase activity of EGFR, or its autocrine activation, thereby increasing the sensitivity of EGFR-positive resistant cells to sorafenib, further demonstrating that EGFR is a potential determinant of sorafenib resistance in HCC cells. Therefore, biological analysis of EGFR, whether directly measuring EGFR expression or activity or detecting its ligands, will help predict the efficacy of sorafenib, which will be a promising personalized treatment option for patients with HCC (Ezzoukhry et al., 2012).

Cancer stem cells (CSCs)

In many solid tumors, a small proportion of cells with progenitor-like features called CSCs or tumor-initiating cells are present (Vidal et al., 2014). Accumulating evidence suggests that CSCs are involved in tumor recurrence, metastasis, and chemoresistance, leading to tumor progression and patient death. Tovar et al. found that tumor tissues from patients with sorafenib-resistant HCC have a higher proportion of CSCs (Arao et al., 2013). Xin et al. conducted a study to test the hypothesis that hepatocarcinoma-derived CSCs are resistant to sorafenib treatment (Xin et al., 2013). The increase in CSC counts was accompanied by reduced apoptosis compared with the findings in the presence of non-CSCs, and the reduction in apoptosis was associated with excessive activation of AKT and ERK. In this study, CSCs were linked to a survival advantage over non-CSCs after sorafenib treatment of tumor cells, resulting in a significant increase in the relative proportion of CSCs in all HCC cell lines tested, and this may be related to liver cancer recurrence after sorafenib treatment (Xin et al., 2013). In addition, Mok et al. (2022) showed that SREBP2-mediated cholesterol biosynthesis is crucial for the increase of hepatic CSCs, and deletion of sterol-regulatory element binding protein 2 (SREBP2) and its chaperone SCAP conferred sensitivity to tyrosine kinase inhibitors in tumor-bearing mice (Li et al., 2022; Mok et al., 2022).

Therefore, the study of the mechanism of drug resistance in CSCs will provide good clinical benefits in sorafenib-resistant patients. The Wnt/β-catenin pathway regulates stem cell proliferation and differentiation, and the Wnt signaling pathway is closely related to drug resistance caused by CSCs and tumor recurrence and metastasis (Lou and Dean, 2007). Histone demethylation has proven essential for the self-renewal/differentiation of stem cells, and the activity of lysine-specific demethylase 1 (LSD1) is required for the appearance of CSCs after long-term sorafenib treatment in patients with HCC. LSD1 can demethylate the monomethyl and dimethyl residues of lysine-4 (H3K4me1 or H3K4me2) on histone H3, thereby inhibiting the expression of several suppressors of β-catenin signaling,

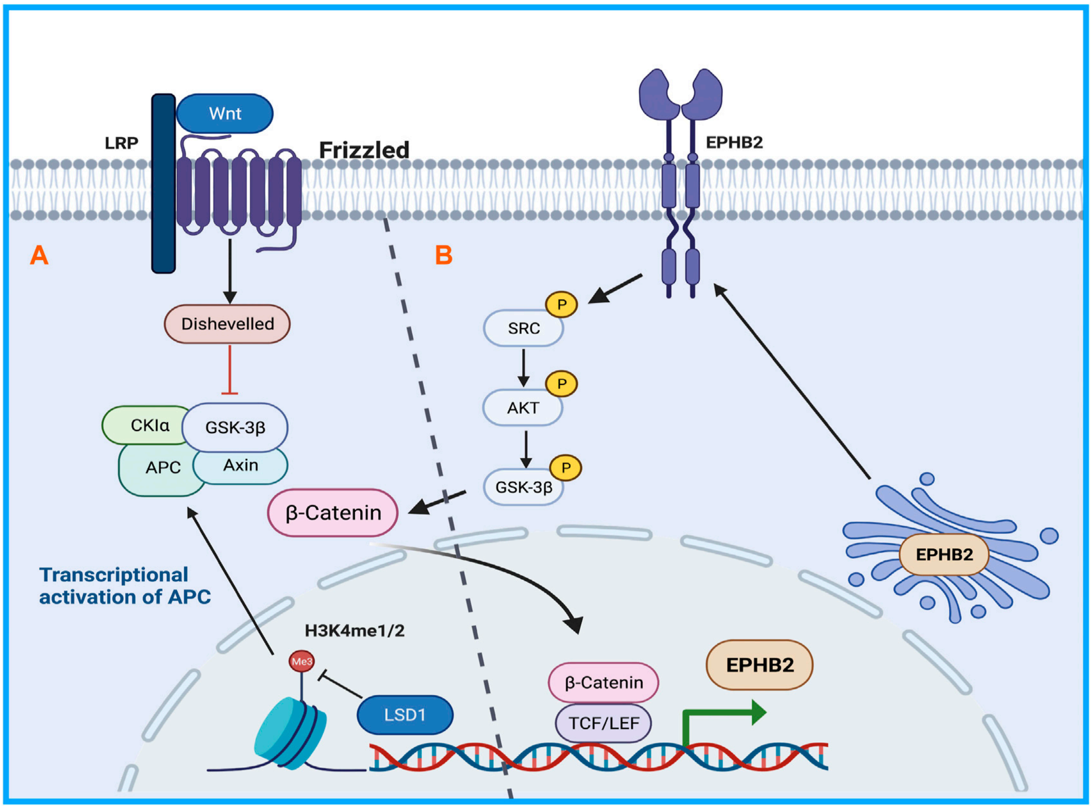

FIGURE 1
The Wnt/β-catenin pathway regulates stem cell proliferation and differentiation. **(A)**: LSD1 is able to demethylate the monomethyl and dimethyl residues of lysine-4 on histone H3, thereby inhibiting the expression of several suppressors of β-catenin signaling promoting β-catenin activation, thereby promoting self-renewal and drug resistance in CSCs. **(B)**: TCF1/EPHB2/β-catenin positive feedback loop regulates cancer stemness and drug resistance.

especially Prickle one and APC in Lgr5⁺ CSCs, and promoting the activation of β-catenin, thereby stimulating self-renewal and drug resistance in Lgr5⁺ CSCs (Lei et al., 2015). Studies illustrated that LSD1 inhibitors can partially restore the sensitivity of resistant cells to sorafenib by inhibiting the Wnt/β-catenin signaling pathway and reducing the self-renewal capacity of CSCs (Huang et al., 2017). In addition, some researchers found that EPHB2 kinase expression is elevated in sorafenib-resistant HCC cells, and this kinase regulates cancer stemness and drug resistance through the TCF1/EPHB2/β-catenin positive feedback loop. In immunocompetent mouse models, targeting EPHB2 with rAAV-8-shEPHB2 (EPHB2 inhibitor) inhibited HCC tumor growth and sensitized HCC cells to sorafenib (Leung et al., 2021), indicating that targeting tumor cell stemness may be a feasible therapeutic strategy against sorafenib resistance in HCC(Figure 1).

Acquired drug resistance

PI3K/AKT and MAPK/ERK signaling pathways

Since 2007, preclinical studies have elucidated that many growth factors, including hepatocyte growth factor, insulin-like growth factor, and fibroblast growth factor, play vital roles in sorafenib resistance by activating the PI3K/AKT and MAPK/ERK pathways (Arao et al., 2013; Nishida et al., 2015; Xu et al., 2016; Han et al., 2017). The PI3K/AKT/mTOR signaling pathway is one of the most common dysregulated pathways in human cancer, and it controls key cellular processes, such as metabolism, motility, growth, and proliferation (Janku et al., 2018). Previous studies illustrated that acquired resistance to sorafenib in HCC may be caused by compensatory activation of the PI3K/AKT pathway. AKT activation promotes tumor cell proliferation and apoptosis resistance; thus, inhibition of AKT activity reverses acquired resistance to sorafenib in HCC (Chen et al., 2011). Similarly, such compensatory signaling activation included the MAPK/ERK pathway, and if only the ERK cascade or AKT pathway is activated after sorafenib treatment, cancer cells could evade apoptosis (Aksamitiene et al., 2012). Han et al. found using sorafenib-resistant HCC cells generated from sorafenib-sensitive human HCC cells that continuous exposure to sorafenib increased hepatocyte growth factor production and c-Met phosphorylation, leading to the activation of AKT and ERK pathways (Han et al., 2017). Dual inhibition of Akt and c-Met by the inhibitors MK2206 and capmatinib, respectively, could inhibit the proliferation of sorafenib-resistant HCC cells *in vitro* and sorafenib-resistant HCC xenografts in mice (Han et al., 2017). Tivantinib, a highly selective inhibitor of c-MET, has

demonstrated its value in different tumors (Santoro et al., 2013; Calles et al., 2015; Moosavi et al., 2021). It has been shown that by inhibiting the expression of EMT and MDR (multidrug resistance) related genes, the combination of tivatinib slowed the clearance of sorafenib in HCC cells and enhanced the anti-tumor effect of sorafenib (Aoyama et al., 2014; Gao et al., 2019). Zhang et al. (2021a) found that 2-(1′H-indole-3′-carbonyl)-thiazole-4-carboxylic acid methyl ester, a ligand of the aryl hydrocarbon receptor, could inhibit the growth and induce the apoptosis of LM3 sorafenib-resistant HCC cells, and these effects were related to inhibition of the PI3K/AKT pathway. Therefore, inhibiting PI3K/AKT and MAPK/ERK alone or in combination can effectively restore the sensitivity of resistant cells to sorafenib.

Epithelial–mesenchymal transition (EMT)

EMT is a key step in the process of cancer cell metastasis, and it is widely involzved in chemoresistance in a variety of tumors (Du and Shim, 2016). During treatment, sorafenib can inhibit the occurrence of EMT, whereas sorafenib-resistant HCC cells exhibit higher metastatic potential, which may be attributable to EMT activation (Chow et al., 2013). It has been observed that the expression of Snail, a core factor of EMT, is increased in sorafenib-resistant HCC cells, leading to EMT characteristics in sorafenib-resistant HCC cells (Zhao et al., 2021a). As the detailed process between EMT and sorafenib resistance remains complex and elusive, researchers have focused on the exact component driving EMT. It has been shown that the transcription factor E26 transformation–specific-1(Ets-1), which is commonly increased in sorafenib-resistant tumor cells, promotes key EMT gene expression (Gluck et al., 2019). Further studies by Vishnoi et al. (2022) identified antioxidant protein GPX2 as a downstream mediator of Ets-1 induced sorafenib resistance, and knockdown of GPX2 expression significantly increased the sensitivity of sorafenib resistant cells to sorafenib (Vishnoi et al., 2022). The results indicate the activation of a novel Ets-1-GPX2 signaling axis in sorafenib resistant cells, targeting which might successfully antagonize resistance (Vishnoi et al., 2022). Tan et al. (2019) found that tumor necrosis factor (TNF-α), a key cellular inflammatory factor, can promote sorafenib resistance in HCC cells by inducing EMT through activating the TNF-α/NF-κB/EMT pathway, and inhibition of TNF-α expression using ulinastatin significantly enhanced the anti-tumor effect of sorafenib in HCC cells with high TNF-α expression. The results suggest that TNF-α, an upstream factor driving EMT, can be used as a predictor of sorafenib sensitivity in patients with HCC. Recently, some studies reported that the phenotypic changes of EMT are caused by epigenetic changes (Nieto et al., 2016). Galle et al. (2020) demonstrated that DNA methylation-driven EMT is the basis of sorafenib treatment resistance in patients with advanced HCC. EMT in blood can be monitored by assessing the DNA methylation status of CpG sites of EMT genes to predict sorafenib resistance (Galle et al., 2020).

Glycolysis

This reprogrammed cancer metabolism is characterized by enhanced glycolysis and inhibition of oxidative phosphorylation, known as the Warburg effect (Stine et al., 2022). It has been reported that the bioenergetic propensity to utilize glycolysis is closely related to sorafenib resistance; thus, inhibiting glycolysis and activating oxidative phosphorylation can overcome intrinsic and acquired sorafenib resistance in HCC cells (Shen et al., 2013). Rate-limiting enzymes in glycolysis, such as 6-phosphofructose-1-kinase, pyruvate kinase, and hexokinase, are activated in sorafenib-resistant HCC cells. Consequently, inhibiting these enzymes to overcome sorafenib resistance is considered an effective treatment strategy (Li et al., 2017; Feng et al., 2019). Further exploration of the relationship between glycolysis and sorafenib resistance revealed that HIF-1a plays an important role in regulating glycolysis and apoptosis. HIFs mediate the primary transcriptional response under hypoxic stress and enhance the expression of several genes involved in glycolysis (Semenza, 2013). Therefore, HIF-1a inhibition might represent a strategy to overcome sorafenib resistance. Meanwhile, the PI3K/Akt pathway is closely related to glucose metabolism in tumor cells, and it is also involved in the regulation of HIF-1α expression, indicating that the PI3K/Akt/HIF-1α pathway plays a key role in the synergistic effect of hypoxia and the Warburg effect. Zhang et al. (2020) found that microbial-derived staphylococcal superantigen-like protein six could inhibited glycolysis by blocking the activation of PI3K/Akt/HIF-1 by CD47 to enhance the sensitivity of HCC cells to sorafenib (Figure 2A). In addition, glycolysis is closely related to CSCs. In HCC, compared with the effects of non-CSCs, CSCs exhibited a higher rate of glycolysis and higher expression of glycolytic genes; thus, inhibiting glycolysis could reduce the number of CSCs to overcome sorafenib resistance (Schieber and Chandel, 2013; Shen et al., 2015). Bi et al. (2021) found that loss of the histone deacetylase HDAC11 increased the transcription of LKB1, a serine/threonine kinase, by promoting histone acetylation in the LKB1 promoter region, which activated the AMPK signaling pathway and inhibited the glycolytic pathway, resulting in the inhibition of tumor cell stemness and the improvement of sorafenib resistance (Figure 2B). Therefore, the resistance profile of HCC cells to sorafenib can be effectively improved by regulating the glycolytic level of tumor cells.

Autophagy

Autophagy is the main intracellular degradation system that eliminates damaged intracellular organelles and misfolded proteins (Zhao et al., 2021b). It has been documented that autophagy has a paradoxical relationship in the development of resistance to sorafenib treatment in HCC (Zhong et al., 2016; Yazdani et al., 2019). On the one hand, researchers found that sorafenib protected tumor cells by activating autophagy in parental HCC cells. Some researchers found that sorafenib resistance associated with CD24 (CSC marker) is accompanied by the activation of autophagy, and resistance can be blocked by inhibiting autophagy using pharmacological inhibitors or knocking out autophagy-related genes. In further studies, investigated revealed that CD24 overexpression leads to increased PP2A protein production and induces inactivation of the mTOR/AKT pathway, thereby increasing autophagy levels. These experimental results indicate that CD24 can lead to sorafenib resistance progression by activating autophagy in hepatoma cells (Lu et al., 2018). Another experiment also confirmed that in HCC, ANXA3-mediated autophagy activation and attenuation of the PKCδ/p38-dependent apoptotic signaling pathway are involved in the development of

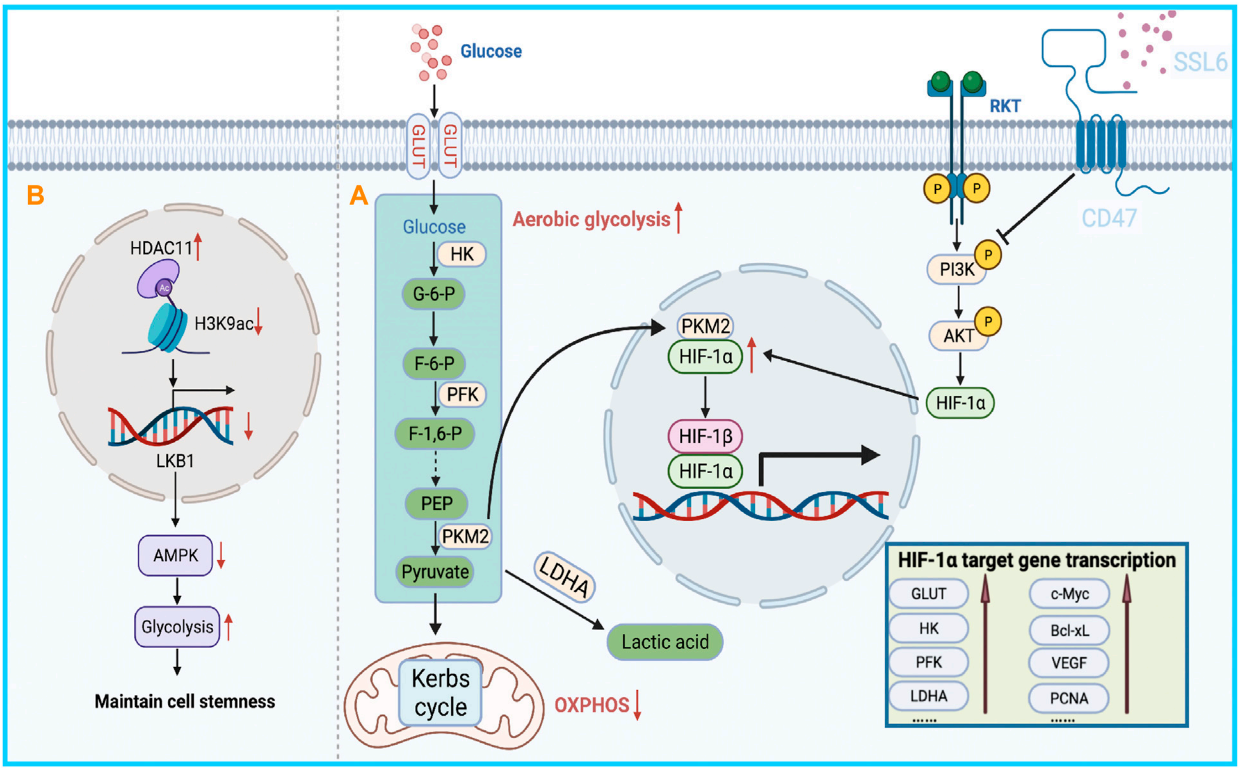

FIGURE 2
Bioenergetic propensity of HCC cells to utilize glycolysis is associated with sorafenib resistance. **(A)**: HIFs mediate the primary transcriptional response to hypoxic stress and promote the expression of glycolysis-regulating enzymes. **(B)**: HDAC11 inhibits the transcription of LKB1 by regulating histone acetylation in the promoter region of LKB1, thereby blocking AMPK signaling and inhibiting the glycolytic pathway, which in turn maintains tumor cell stemness.

sorafenib resistance, and HCC cells can be resensitized to sorafenib by inhibiting the expression of ANXA3 protein (Tong et al., 2018).

On the other hand, in resistant cell lines, the protective effect of autophagy could be switched to a role in promoting cell death. Because continuous drug exposure can induce unbalanced apoptotic pathways, it leads to cell resistance to apoptosis (Neophytou et al., 2021). Autophagy, as an adaptive response, switches from cytoprotective activity to pro-death functioning when apoptotic signals decay (Su et al., 2013; Zhai et al., 2014; Booth et al., 2020; Chen et al., 2021). Therefore, researchers found that inhibition of autophagy further reduced sorafenib sensitivity in sorafenib-resistant HCC cells, and conversely, inhibition of Akt induced a switch of autophagy from cytoprotective to pro-death mechanisms, thereby reversing acquired resistance to sorafenib (Neophytou et al., 2021). Because of the dual role of autophagy in the process of sorafenib resistance, additional caution is needed for clinical drugs that induce autophagy.

Non-coding RNAs

MicroRNAs (miRNAs) are sequences with an average length of 22 nucleotides that regulate target mRNA expression by binding to a short complementary sequence in the 3′-UTR region of mRNAs (Sun and Lai, 2013). In mammalian cells, miRNAs complex with Argonaute and Dicer to form RNA-induced silencing complex and guide them to cleave complementary mRNAs to achieve gene silencing, thereby inhibiting protein synthesis (Meister and Tuschl, 2004). Recent studies illustrated that the differential expression of miRNAs is closely related to sorafenib resistance in HCC, and most miRNAs exhibit lower expression in resistant tumor tissues than in normal tissues (Wei et al., 2019). For instance, miR-622 inhibits the expression of KRAS, leading to inhibition of the RAF/MAPK and PI3K/AKT pathways, suppression of HCC growth, and enhancement of sorafenib sensitivity (Dietrich et al., 2018). Therefore, miR-622 expression can be used as an auxiliary diagnostic tool to predict the response to sorafenib treatment in patients with HCC. Kabir et al. found that miR-7 is a potent tumor suppressor in human HCC, and TYRO3 is a novel functional target of miR-7 (Kabir et al., 2018). TYRO3 is a member of the TAM family of RTKs, and aberrant expression of the TYRO3/PI3K/AKT signaling pathway is a novel mechanism of acquired resistance to sorafenib in HCC. Experimental data illustrated that miR-7 overexpression could effectively silence TYRO3 expression in sorafenib-sensitive and sorafenib-resistant Huh-7 cells, thereby overcoming sorafenib resistance in HCC caused by abnormal TYRO3 expression (Kabir et al., 2018). Ji et al. (2020) found that miR-486-3p was significantly downregulated in sorafenib-resistant

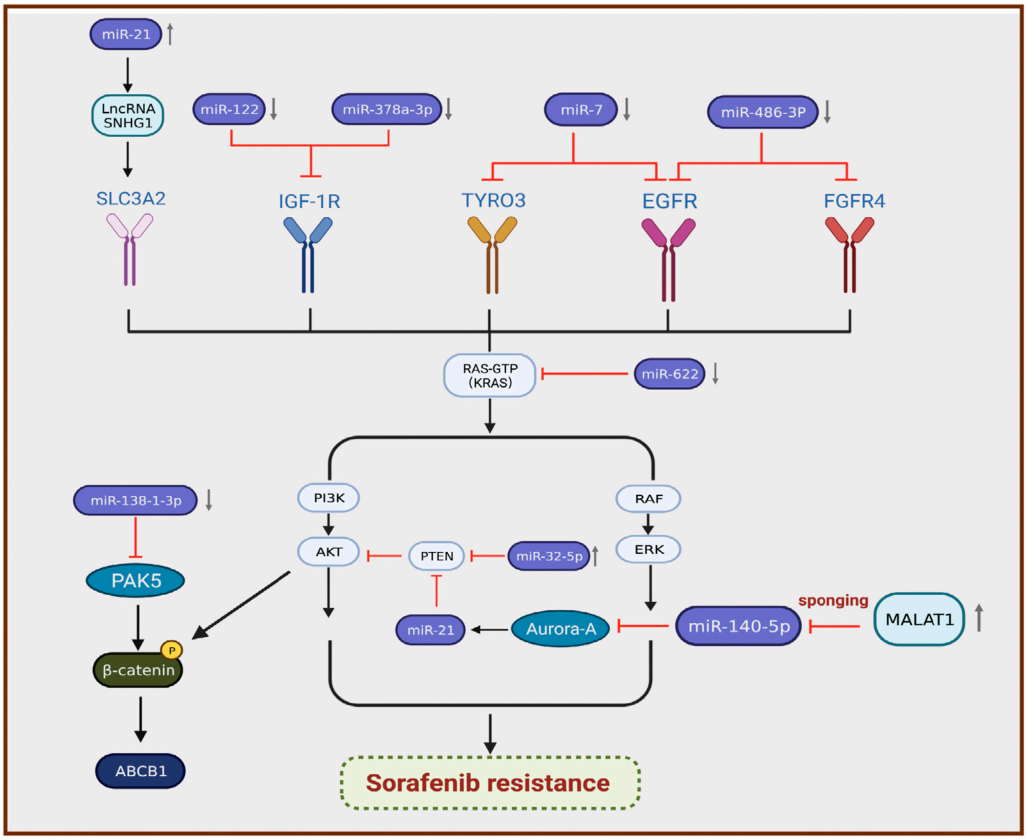

FIGURE 3
Differential expression of miRNAs is associated with the development of sorafenib resistance in hepatocellular carcinoma.

HCC cell lines, further validating FGFR4 and EGFR as targets of miR-486-3p, and overexpression of miR-486-3p in combination with sorafenib could significantly inhibit tumor growth in a sorafenib resistance model. Li et al. (2021) detected significant downregulation of miR-138-1-3p and upregulation of PAK5 in sorafenib-resistant HCC cell lines. They found that PAK5 elevated the phosphorylation and nuclear translocation of β-catenin, thereby increasing the transcriptional activity of the multidrug resistance protein ABCB1, indicating that miR-138-1-3p mediates sorafenib resistance by negatively regulating PAK5 (Li et al., 2021). IGF-1 receptor (IGF-1R) is a major member of the tyrosine protein kinase receptor family that plays an important role in maintaining the malignant phenotype and anti-apoptosis of tumors. Overexpression of IGF-1R and its ligand IGF-1 is associated with tumor progression. Studies illustrated that IGF signaling is enriched in tumors with acquired resistance to sorafenib (Tovar et al., 2017). Recently, two groups of researchers demonstrated that miR-122 and miR-378a-3p negatively regulated IGF-1R expression. Xu et al. (2016) found that IGF-1R could be activated by the ectopic downregulation of miR-122 to counteract sorafenib-induced apoptosis, thereby inducing sorafenib resistance. Similarly, Lin et al. (2020) confirmed that decreased XPO5 expression prevented the maturation of miR-378a-3p, which resulted in overexpression of IGF-1R and counteracted the effect of sorafenib-induced apoptosis. Mechanistically, downregulation of IGF-1R by miR-122 and miR-378a-3p contributes to activation of the RAS/RAF/ERK signaling pathway, which is associated with drug resistance (Xu et al., 2016; Lin et al., 2020)(Figure 3).

By contrast, certain miRNAs are overexpressed in HCC. Fu et al. (2018) revealed that miR-32-5p was significantly upregulated in multidrug-resistant cell lines and positively correlated with low PTEN expression as well as poor prognosis. Overexpression of miR-32-5p activates the PI3K/Akt pathway by inhibiting PTEN and further induces multidrug resistance by regulating angiogenesis and EMT (Fu et al., 2018). Li et al. (2019) found that sorafenib induced the translocation of miR-21 to the nucleus and promoted the expression of the lncRNA small nucleolar RNA host gene 1, resulting in upregulation of SLC3A2 and activation of the Akt pathway, which is involved in the progression of sorafenib resistance. MiRNAs can also interact with lncRNAs to participate in the progression of sorafenib resistance. Fan et al. (2020) verified that the lncRNA MALAT1 regulates Aurora-A expression through the sponge miR-140-5p, which promotes sorafenib resistance in HCC cells (Figure 3). MALAT1 therefore has the potential to serve as a novel target for prognostic prediction and therapeutic strategies in patients with HCC treated with sorafenib.

Several miRNAs are widely involved in the development of sorafenib resistance in patients with HCC by regulating the differential expression of sorafenib-targeted kinases. Recent studies

demonstrated that miRNAs can be used as tissue-specific biomarkers to predict sorafenib resistance in patients with HCC, and the sensitivity of resistant cells to sorafenib can be effectively improved by artificially altering the content of miRNAs within HCC cells. Simultaneously, the involvement of miRNAs in regulating sorafenib resistance in HCC is a multi-level and multi-target process, and thus, the synergistic regulation of multiple miRNAs can be considered when studying the expression of miRNAs within HCC cells. In studying strategy to reverse sorafenib resistance, miRNAs represent an important link that must be considered, and studies targeting miRNAs will yield great benefits for the prognosis of patients with sorafenib-resistant HCC.

Apoptosis resistance and deregulated cell cycle control

Evasion of apoptosis is a common feature of cancer cells that is tightly associated with drug resistance (Shahar and Larisch, 2020). Cancer cells overexpress many proteins that play important roles in resisting activation of the apoptotic cascade, such as Bcl-2, Bcl-xL, and Mcl-1 (Mohammad et al., 2015). Dudgeon et al. found that sorafenib was able to kill cancer cells by activating PUMA (an apoptotic modulator upregulated by p53) (Dudgeon et al., 2012). As a large subclass of the Bcl-2 protein family, PUMA, a BH3-domain only protein, is a key initiator of apoptosis in cancer cells (Edwards et al., 2013). PUMA deficiency abolished apoptosis and caspase activation induced by sorafenib, whereas BH3 analogs enhanced the anti-cancer effect of sorafenib and restored the sensitivity of resistant cells to sorafenib (Dudgeon et al., 2012). As an oncogenic driver in HCC, FGF19, with its main receptor FGFR4, is highly expressed in primary HCC, and its new role in sorafenib resistance was reported (Repana and Ross, 2015). By overexpressing FGF19, tumor cells inhibit ROS generation and apoptosis induced by sorafenib. Importantly, targeting the FGF19/FGFR4 axis by administering ponatinib, a third-generation inhibitor for chronic myelogenous leukemia treatment, can overcome resistance to sorafenib in HCC by enhancing ROS-related apoptosis (Gao et al., 2017). These findings suggest that exploring apoptotic mechanisms provides a theoretical basis for improving cell sensitivity to targeted therapy.

Furthermore, dysregulation of cell cycle control is a hallmark of cancer, and overexpression of cyclins is usually closely related to tumorigenesis progression (Suski et al., 2021). Many studies demonstrated that synergistic use of cell cycle inhibitors enhanced sorafenib anticancer activity as well as partially antagonize multidrug resistance (Reiter et al., 2019; Lee et al., 2020; Yu et al., 2020). Hsu et al. found that the regulation of the E2F1–Rb–cyclin E1 complex might play a crucial role in mediating sorafenib resistance in HCC cells, and depletion of cyclin E1 expression reversed sorafenib resistance in HCC cells in terms of cell growth and apoptosis induction (Hsu et al., 2016). In addition, the combination of sorafenib and CDK inhibitors might improve the efficacy of sorafenib in the treatment of HCC (Hsu et al., 2016).

Lenvatinib resistance

Lenvatinib is an oral inhibitor of multiple RTKs including VEGFR1–3, FGFR1–4, platelet PDGFRα, RET, and KIT (Matsui et al., 2008). It became the second approved first-line treatment for patients with advanced HCC in 2018, and its efficacy against some RTKs was superior to that of sorafenib (Kudo et al., 2018; Matsuki et al., 2018). However, similar to sorafenib, lenvatinib initially controls tumors well, but resistance gradually develops over time.

In contrast to classical cytotoxic agents, lenvatinib targets specific molecular and cancer signaling pathways. Therefore, the diversity of genetic drivers may have a great impact on the anti-tumor effect of lenvatinib, and Ao et al. (2021) identified three angiogenic cytokines, VEGF, platelet-derived growth factor-AA, and angiogenin, as vital factors for lenvatinib-resistance *via* tumor angiogenesis. Meanwhile, as targets of lenvatinib, kinases such as FGFR1, FGFR4, and VEGFR are associated with lenvatinib resistance, and inhibiting these target kinases *via* certain alkaloid extracts could improve the sensitivity of lenvatinib (Zhao et al., 2021c; Zhao et al., 2021d; Shi et al., 2021). For instance, the alkaloid Oxysophocarpine extracted from seaweeds can inhibit the proliferation and induce apoptosis of HCC cells with FGFR1 overexpression (Zhao et al., 2021c). Similarly to sorafenib resistance, non-coding RNAs are also involved in the development of lenvatinib resistance, and Yu et al. (2021) confirmed that the lncRNA MT1JP was upregulated to inhibit apoptotic signaling pathways in LR-HCC cells, thereby reducing the sensitivity of hepatocytes to lenvatinib. Recently, Duan et al. (2022) first reported that long non-coding RNA XIST can promote lenvatinib resistance in hepatocellular carcinoma cells through epigenetic inhibition of NOD2 (Nucleotide-binding oligomerization domain 2) (Duan et al., 2022). Mechanistically, lncXIST is able to bind to the histone modifying enzyme EZH2, which acts as a core subunit of the PRC2 complex (Polycomb Repressive Complex 2) and promotes transcriptional silencing by catalyzing trimethylation of histone H3K27, thus negatively regulating NOD2 expression (Liu et al., 2021; Duan et al., 2022). EZH2 has been reported to be overexpressed in HCC and associated with poor prognosis (Bae et al., 2022). The results of this study further suggest EZH2 as a target to overcome lenvatinib resistance in HCC cells.Furthermore, Zhang et al. (2021b) found that the circRNA circMED27 was significantly upregulated in HCC serum, and it acts as competitive endogenous RNA for miR-655-3p. It act as a sponge to adsorb miR-655-3p and then upregulate the expression of ubiquitin-specific peptidase 28 (USP28), promoting the resistance of HCC cells to lenvatinib (Zhang et al., 2021b). Research into the resistance mechanism of lenvatinib, as the second first-line HCC treatment, will benefit the treatment of patients with HCC.

Resistance to other kinase inhibitors

After many patients failed treatment with sorafenib because of multi-mechanism resistance, regorafenib was approved by the FDA in 2017 for the second-line treatment of patients with unresectable HCC (Bruix et al., 2017; Duffy and Greten, 2017). However, as a kinase inhibitor, the efficacy of regorafenib declines because of inevitable drug resistance. Therefore, elucidating the potential mechanism of regorafenib resistance is crucial for the use of regorafenib in the treatment of HCC. Pin1, a unique phosphorylation-specific peptidyl-prolyl cis-

trans isomerase, is a common regulator of a variety of oncogenic signaling networks, and it was identified as a key isomerase in regulating HCC progression (Wei et al., 2015; Pu et al., 2018). Wang et al. (2019). demonstrated that inhibition of Pin1 can reverse acquired resistance to regorafenib in HCC in part by inhibiting EMT through the Gli1/Snail/E-cadherin pathway. Their study revealed for the first time the molecular mechanism of regorafenib resistance in HCC, suggesting that Pin1 inhibitors will be an alternative treatment class for the treatment of aggressive and regorafenib-resistant HCC (Wang et al., 2019). Recently, many findings illustrated that overexpression of sphingosine kinase 2 (SphK2) is associated with drug resistance in tumor cells (Liu et al., 2016). Shi et al. (2020) first demonstrated that SphK2/S1P is a key regulator mediating regorafenib resistance to HCC through NF-κB and STAT3 activation. Thus, ABC294640, a selective inhibitor of SphK2, exhibited high potential to increase the sensitivity of regorafenib-resistant HCC cells to the drug (Shi et al., 2020). Karabicici et al. (2021) found that HCC tumors with abnormal Wnt/β-catenin activation may have higher intrinsic regorafenib resistance (Karabicici et al., 2021). After this, Wang et al. (2022) found that silencing TOP2A, a key pro-oncogene in a variety of tumors, blocked the EMT process and reversed acquired resistance to regorafenib through the Wnt-β-catenin pathway (Wang et al., 2022).In addition, regorafenib resistant cells have enhanced TGF-β signaling activity and significantly higher *in vivo* migration ability, which could be reversed upon TGFβ-R1 inhibition (Karabicici et al., 2021). Therefore, the combined use of TGF-β pathway inhibitors and regorafenib is a promising method for sensitization and prevention of tumor recurrence in patients with HCC with acquired regorafenib resistance. Recently, some preclinical findings suggest that regorafenib exhibits anti-immunosuppressive properties as an anti-angiogenic agent (Schmittnaegel and De Palma, 2017; Arai et al., 2019). For example, regorafenib can promote anti-tumor immunity by regulating macrophages and increasing the proliferation and activation of $CD8^+$T cells, so the combination of regorafenib with immune checkpoint inhibitors can be regarded as a new dosing strategy, which may delay the development of its resistance (Granito et al., 2021).

Cabozantinib is a small-molecule kinase inhibitor with potent activity against MET, VEGFR2, RET, KIT, AXL, and FLT3. These kinases have been implicated in HCC progression and the development of resistance to sorafenib (Yakes et al., 2011). Cabozantinib was approved in 2019 for patients with advanced HCC who have been treated with sorafenib (Abou-Alfa et al., 2018). c-MET plays a key role in the occurrence and development of HCC, which is related to HCC cell proliferation, survival, and invasiveness, angiogenesis and the development of resistance to chemotherapeutic drugs (Venepalli and Goff, 2013; Bouattour et al., 2018). c-MET is the main target of the anti-tumor activity of cabozantinib. However, Gao et al. found that HCC cells with low c-Met levels exhibited primary resistance to c-MET inhibitors, and the combination of cabozantinib and the mTOR inhibitor rapamycin exerted synergistic inhibitory effects on cell proliferation and tumor growth in resistant cells (Gao et al., 2021; Shang et al., 2021). Therefore, these results suggest that the development of cabozantinib resistance can be partially avoided using rational combinations.

Possible strategies

Systemic therapy plays an important role in the treatment of liver cancer. On the one hand, patients with advanced HCC miss the opportunity for surgical resection or local treatment. On the other hand, the majority of patients with HCC have to receive systemic treatment after resection treatment because of the high recurrence rate of HCC (Villanueva, 2019). It is worth mentioning that before sorafenib was approved as the first-line treatment for advanced HCC in 2008, there were no curative treatments for patients with advanced HCC.

Although small-molecule targeted drugs such as sorafenib prolong the survival of patients with advanced HCC, the occurrence of drug resistance greatly reduces their clinical benefits. It is gratifying that with the development of molecular biology, the mechanism of kinase drug resistance has gradually been revealed, thereby providing key insights into clinical treatment (Kannaiyan and Mahadevan, 2018). First, in the case of sorafenib, because of the genetic heterogeneity of tumors, many patients exhibit primary resistance characteristics during the initial treatment. Thus, it is possible to predict the efficacy of sorafenib by defining molecular markers related to resistance through tumor genome sequencing technology in clinical practice. These novel molecular markers might include EGFR and its downstream molecules or cellular markers affecting tumor stemness. Second, patients who develop acquired resistance after sorafenib treatment can be treated by adjusting the dose or changing to another targeted drug. For example, immune checkpoint inhibitors can be used as alternative treatment options. The combination of atezolizumab and the antiangiogenic agent bevacizumab prolonged OS versus sorafenib monotherapy in patients with advanced HCC (Hack et al., 2020).

In addition, drug intervention against some specific targets can also be used to resensitize tumors to sorafenib. The Akt inhibitor GDC0068 can reverse acquired resistance to sorafenib by switching autophagy from cytoprotective to pro-death activity (Zhai et al., 2014). TNF-α/NF-κB/EMT signaling inhibition using ulinastatin overcomes sorafenib resistance in HCC (Tan et al., 2019).

Traditional cancer treatments are based on the continuous administration of fixed doses of single or multiple drugs, using the MTD (maximum tolerated dose) to kill as many cancer cells as possible to obtain the greatest therapeutic effect. However, increasing evidence suggests that treatments aimed at eliminating susceptible subpopulations result in altered tumor microenvironment favoring resistant subpopulations, which enhances the probability and rate of resistance emergence (Chatterjee and Bivona, 2019). Increasing research has focused on dose strategies to combat resistance in tumor progression in combination therapy, and both extremes of too high and too low doses may unnecessarily accelerate the spread of resistance (Kouyos et al., 2014; Vakil and Trappe, 2022). Some results suggest that maintaining a low-dose treatment strategy is advantageous when the

size of the patient's tumor is tolerable, as it allows the susceptible clonal population to survive and compete with the resistant subpopulation to prevent the resistant population from propagating uncontrollably and taking over the entire tumor, thus having more feared consequences for the patient (Vakil and Trappe, 2021). In addition, in situations where the patient's immune response increases over time, delaying the emergence of resistance may provide sufficient time for immunity to help prevent resistance. These interesting findings suggest whether the administered dose of TKIs can be minimized to delay the development of drug resistance phenomenon under the premise of maintaining the survival status of patients.

In conclusion, in this paper, we reviewed the resistance mechanisms of small-molecule kinase inhibitors in the treatment of HCC and corresponding improved strategies, hoping to improve the outcomes of patients with HCC.

Author contributions

LJ, LiL, and YoL analysed most of the data, and wrote the initial draft of the paper; YaL and SY developed the idea for the study; all authors contributed to the writing and revisions.

Funding

This study is supported by the National Natural Science Foundation of China (82230067), the Guangdong Provincial Key Laboratory of Tumor Interventional Diagnosis and Treatment (2021B1212040004).

Conflict of interest

The authors declare that the research was conducted in the absence of any commercial or financial relationships that could be construed as a potential conflict of interest.

Publisher's note

All claims expressed in this article are solely those of the authors and do not necessarily represent those of their affiliated organizations, or those of the publisher, the editors and the reviewers. Any product that may be evaluated in this article, or claim that may be made by its manufacturer, is not guaranteed or endorsed by the publisher.

References

Abou-Alfa, G. K., Meyer, T., Cheng, A. L., El-Khoueiry, A. B., Rimassa, L., Ryoo, B. Y., et al. (2018). Cabozantinib in patients with advanced and progressing hepatocellular carcinoma. *N. Engl. J. Med.* 379 (1), 54–63. doi:10.1056/NEJMoa1717002

Aksamitiene, E., Kiyatkin, A., and Kholodenko, B. N. (2012). Cross-talk between mitogenic Ras/MAPK and survival PI3K/Akt pathways: A fine balance. *Biochem. Soc. Trans.* 40 (1), 139–146. doi:10.1042/BST20110609

Ao, J., Chiba, T., Shibata, S., Kurosugi, A., Qiang, N., Ma, Y., et al. (2021). Acquisition of mesenchymal-like phenotypes and overproduction of angiogenic factors in lenvatinib-resistant hepatocellular carcinoma cells. *Biochem. Biophys. Res. Commun.* 549, 171–178. doi:10.1016/j.bbrc.2021.02.097

Aoyama, A., Katayama, R., Oh-Hara, T., Sato, S., Okuno, Y., and Fujita, N. (2014). Tivantinib (ARQ 197) exhibits antitumor activity by directly interacting with tubulin and overcomes ABC transporter-mediated drug resistance. *Mol. Cancer Ther.* 13 (12), 2978–2990. doi:10.1158/1535-7163.MCT-14-0462

Arai, H., Battaglin, F., Wang, J., Lo, J. H., Soni, S., Zhang, W., et al. (2019). Molecular insight of regorafenib treatment for colorectal cancer. *Cancer Treat. Rev.* 81, 101912. doi:10.1016/j.ctrv.2019.101912

Arao, T., Ueshima, K., Matsumoto, K., Nagai, T., Kimura, H., Hagiwara, S., et al. (2013). FGF3/FGF4 amplification and multiple lung metastases in responders to sorafenib in hepatocellular carcinoma. *Hepatology* 57 (4), 1407–1415. doi:10.1002/hep.25956

Bae, A. N., Jung, S. J., Lee, J. H., Lee, H., and Park, S. G. (2022). Clinical value of EZH2 in hepatocellular carcinoma and its potential for target therapy. *Med. Kaunas.* 58 (2), 155. doi:10.3390/medicina58020155

Blivet-Van Eggelpoël, M. J., Chettouh, H., Fartoux, L., Aoudjehane, L., Barbu, V., Rey, C., et al. (2012). Epidermal growth factor receptor and HER-3 restrict cell response to sorafenib in hepatocellular carcinoma cells. *J. Hepatol.* 57 (1), 108–115. doi:10.1016/j.jhep.2012.02.019

Bi, L., Ren, Y., Feng, M., Meng, P., Wang, Q., Chen, W., et al. (2021). HDAC11 regulates glycolysis through the LKB1/AMPK signaling pathway to maintain hepatocellular carcinoma stemness. *Cancer Res.* 81 (8), 2015–2028. doi:10.1158/0008-5472.CAN-20-3044

Booth, L. A., Roberts, J. L., and Dent, P. (2020). The role of cell signaling in the crosstalk between autophagy and apoptosis in the regulation of tumor cell survival in response to sorafenib and neratinib. *Semin. Cancer Biol.* 66, 129–139. doi:10.1016/j.semcancer.2019.10.013

Bouattour, M., Raymond, E., Qin, S., Cheng, A. L., Stammberger, U., Locatelli, G., et al. (2018). Recent developments of c-Met as a therapeutic target in hepatocellular carcinoma. *Hepatology* 67 (3), 1132–1149. doi:10.1002/hep.29496

Bruix, J., Qin, S., Merle, P., Granito, A., Huang, Y. H., Bodoky, G., et al. (2017). Regorafenib for patients with hepatocellular carcinoma who progressed on sorafenib treatment (RESORCE): A randomised, double-blind, placebo-controlled, phase 3 trial. *Lancet* 389 (10064), 56–66. doi:10.1016/S0140-6736(16)32453-9

Calles, A., Kwiatkowski, N., Cammarata, B. K., Ercan, D., Gray, N. S., and Jänne, P. A. (2015). Tivantinib (ARQ 197) efficacy is independent of MET inhibition in non-small-cell lung cancer cell lines. *Mol. Oncol.* 9 (1), 260–269. doi:10.1016/j.molonc.2014.08.011

Chatterjee, N., and Bivona, T. G. (2019). Polytherapy and targeted cancer drug resistance. *Trends Cancer* 5 (3), 170–182. doi:10.1016/j.trecan.2019.02.003

Chen, K. F., Chen, H. L., Tai, W. T., Feng, W. C., Hsu, C. H., Chen, P. J., et al. (2011). Activation of phosphatidylinositol 3-kinase/Akt signaling pathway mediates acquired resistance to sorafenib in hepatocellular carcinoma cells. *J. Pharmacol. Exp. Ther.* 337 (1), 155–161. doi:10.1124/jpet.110.175786

Chen, S., Du, Y., Xu, B., Li, Q., Yang, L., Jiang, Z., et al. (2021). Vaccinia-related kinase 2 blunts sorafenib's efficacy against hepatocellular carcinoma by disturbing the apoptosis-autophagy balance. *Oncogene* 40 (19), 3378–3393. doi:10.1038/s41388-021-01780-y

Chow, A. K., Ng, L., Lam, C. S., Wong, S. K., Wan, T. M., Cheng, N. S., et al. (2013). The Enhanced metastatic potential of hepatocellular carcinoma (HCC) cells with sorafenib resistance. *PLoS One* 8 (11), e78675. doi:10.1371/journal.pone.0078675

Dietrich, P., Koch, A., Fritz, V., Hartmann, A., Bosserhoff, A. K., and Hellerbrand, C. (2018). Wild type Kirsten rat sarcoma is a novel microRNA-622-regulated therapeutic target for hepatocellular carcinoma and contributes to sorafenib resistance. *Gut* 67 (7), 1328–1341. doi:10.1136/gutjnl-2017-315402

Du, B., and Shim, J. S. (2016). Targeting epithelial-mesenchymal transition (EMT) to overcome drug resistance in cancer. *Molecules* 21 (7), 965. doi:10.3390/molecules21070965

Duan, A., Li, H., Yu, W., Zhang, Y., and Yin, L. (2022). Long noncoding RNA XIST promotes resistance to lenvatinib in hepatocellular carcinoma cells via epigenetic inhibition of NOD2. *J. Oncol.* 2022, 4537343. doi:10.1155/2022/4537343

Dudgeon, C., Peng, R., Wang, P., Sebastiani, A., Yu, J., and Zhang, L. (2012). Inhibiting oncogenic signaling by sorafenib activates PUMA via GSK3β and NF-κB to suppress tumor cell growth. *Oncogene* 31 (46), 4848–4858. doi:10.1038/onc.2011.644

Duffy, A. G., and Greten, T. F. (2017). Liver cancer: Regorafenib as second-line therapy in hepatocellular carcinoma. *Nat. Rev. Gastroenterol. Hepatol.* 14 (3), 141–142. doi:10.1038/nrgastro.2017.7

Edwards, A. L., Gavathiotis, E., LaBelle, J. L., Braun, C. R., Opoku-Nsiah, K. A., Bird, G. H., et al. (2013). Multimodal interaction with BCL-2 family proteins underlies the proapoptotic activity of PUMA BH3. *Chem. Biol.* 20 (7), 888–902. doi:10.1016/j.chembiol.2013.06.007

Ezzoukhry, Z., Louandre, C., Trécherel, E., Godin, C., Chauffert, B., Dupont, S., et al. (2012). EGFR activation is a potential determinant of primary resistance of hepatocellular carcinoma cells to sorafenib. *Int. J. Cancer* 131 (12), 2961–2969. doi:10.1002/ijc.27604

Fan, L., Huang, X., Chen, J., Zhang, K., Gu, Y. H., Sun, J., et al. (2020). Long noncoding RNA MALAT1 contributes to sorafenib resistance by targeting miR-140-5p/aurora-A signaling in hepatocellular carcinoma. *Mol. Cancer Ther.* 19 (5), 1197–1209. doi:10.1158/1535-7163.MCT-19-0203

Feng, J., Wu, L., Ji, J., Chen, K., Yu, Q., Zhang, J., et al. (2019). PKM2 is the target of proanthocyanidin B2 during the inhibition of hepatocellular carcinoma. *J. Exp. Clin. Cancer Res.* 38 (1), 204. doi:10.1186/s13046-019-1194-z

Fu, X., Liu, M., Qu, S., Ma, J., Zhang, Y., Shi, T., et al. (2018). Exosomal microRNA-32-5p induces multidrug resistance in hepatocellular carcinoma via the PI3K/Akt pathway. *J. Exp. Clin. Cancer Res.* 37 (1), 52. doi:10.1186/s13046-018-0677-7

Galle, E., Thienpont, B., Cappuyns, S., Venken, T., Busschaert, P., Van Haele, M., et al. (2020). DNA methylation-driven EMT is a common mechanism of resistance to various therapeutic agents in cancer. *Clin. Epigenetics* 12 (1), 27. doi:10.1186/s13148-020-0821-z

Gao, C., Wang, S., Shao, W., Zhang, Y., Lu, L., Jia, H., et al. (2021). Rapamycin enhances the anti-tumor activity of cabozantinib in cMet inhibitor-resistant hepatocellular carcinoma. *Front. Med.* 16, 467–482. doi:10.1007/s11684-021-0869-y

Gao, L., Wang, X., Tang, Y., Huang, S., Hu, C. A., and Teng, Y. (2017). FGF19/FGFR4 signaling contributes to the resistance of hepatocellular carcinoma to sorafenib. *J. Exp. Clin. Cancer Res.* 36 (1), 8. doi:10.1186/s13046-016-0478-9

Gao, X., Chen, H., Huang, X., Li, H., Liu, Z., and Bo, X. (2019). ARQ-197 enhances the antitumor effect of sorafenib in hepatocellular carcinoma cells via decelerating its intracellular clearance. *Onco Targets Ther.* 12, 1629–1640. doi:10.2147/OTT.S196713

Gluck, C., Glathar, A., Tsompana, M., Nowak, N., Garrett-Sinha, L. A., Buck, M. J., et al. (2019). Molecular dissection of the oncogenic role of ETS1 in the mesenchymal subtypes of head and neck squamous cell carcinoma. *PLoS Genet.* 15, e1008250. doi:10.1371/journal.pgen.1008250

Granito, A., Forgione, A., Marinelli, S., Renzulli, M., Ielasi, L., Sansone, V., et al. (2021). Experience with regorafenib in the treatment of hepatocellular carcinoma. *Ther. Adv. Gastroenterol.* 14, 17562848211016959. doi:10.1177/17562848211016959

Hack, S. P., Spahn, J., Chen, M., Cheng, A. L., Kaseb, A., Kudo, M., et al. (2020). IMbrave 050: A phase III trial of atezolizumab plus bevacizumab in high-risk hepatocellular carcinoma after curative resection or ablation. *Future Oncol.* 16 (15), 975–989. doi:10.2217/fon-2020-0162

Han, P., Li, H., Jiang, X., Zhai, B., Tan, G., Zhao, D., et al. (2017). Dual inhibition of Akt and c-Met as a second-line therapy following acquired resistance to sorafenib in hepatocellular carcinoma cells. *Mol. Oncol.* 11 (3), 320–334. doi:10.1002/1878-0261.12039

Hsieh, S. Y., He, J. R., Hsu, C. Y., Chen, W. J., Bera, R., Lin, K. Y., et al. (2011). Neuregulin/erythroblastic leukemia viral oncogene homolog 3 autocrine loop contributes to invasion and early recurrence of human hepatoma. *Hepatology* 53 (2), 504–516. doi:10.1002/hep.24083

Hsu, C., Lin, L. I., Cheng, Y. C., Feng, Z. R., Shao, Y. Y., Cheng, A. L., et al. (2016). Cyclin E1 inhibition can overcome sorafenib resistance in hepatocellular carcinoma cells through mcl-1 suppression. *Clin. Cancer Res.* 22 (10), 2555–2564. doi:10.1158/1078-0432.CCR-15-0499

Huang, M., Chen, C., Geng, J., Han, D., Wang, T., Xie, T., et al. (2017). Targeting KDM1A attenuates Wnt/β-catenin signaling pathway to eliminate sorafenib-resistant stem-like cells in hepatocellular carcinoma. *Cancer Lett.* 398, 12–21. doi:10.1016/j.canlet.2017.03.038

Janku, F., Yap, T. A., and Meric-Bernstam, F. (2018). Targeting the PI3K pathway in cancer: Are we making headway? *Nat. Rev. Clin. Oncol.* 15 (5), 273–291. doi:10.1038/nrclinonc.2018.28

Ji, L., Lin, Z., Wan, Z., Xia, S., Jiang, S., Cen, D., et al. (2020). miR-486-3p mediates hepatocellular carcinoma sorafenib resistance by targeting FGFR4 and EGFR. *Cell Death Dis.* 11 (4), 250. doi:10.1038/s41419-020-2413-4

Kabir, T. D., Ganda, C., Brown, R. M., Beveridge, D. J., Richardson, K. L., Chaturvedi, V., et al. (2018). A microRNA-7/growth arrest specific 6/TYRO3 axis regulates the growth and invasiveness of sorafenib-resistant cells in human hepatocellular carcinoma. *Hepatology* 67 (1), 216–231. doi:10.1002/hep.29478

Kannaiyan, R., and Mahadevan, D. (2018). A comprehensive review of protein kinase inhibitors for cancer therapy. *Expert Rev. Anticancer Ther.* 18 (12), 1249–1270. doi:10.1080/14737140.2018.1527688

Karabicici, M., Azbazdar, Y., Ozhan, G., Senturk, S., Firtina Karagonlar, Z., and Erdal, E. (2021). Changes in Wnt and TGF-β signaling mediate the development of regorafenib resistance in hepatocellular carcinoma cell line HuH7. *Front. Cell Dev. Biol.* 9, 639779. doi:10.3389/fcell.2021.639779

Keating, G. M. (2017). Sorafenib: A review in hepatocellular carcinoma. *Target Oncol.* 12 (2), 243–253. doi:10.1007/s11523-017-0484-7

Kouyos, R. D., Metcalf, C. J., Birger, R., Klein, E. Y., Abel, Z., Wiesch, P., et al. (2014). The path of least resistance: Aggressive or moderate treatment? *Proc. Biol. Sci.* 281 (1794), 20140566. doi:10.1098/rspb.2014.0566

Kudo, M., Finn, R. S., Qin, S., Han, K. H., Ikeda, K., Piscaglia, F., et al. (2018). Lenvatinib versus sorafenib in first-line treatment of patients with unresectable hepatocellular carcinoma: A randomised phase 3 non-inferiority trial. *Lancet* 391 (10126), 1163–1173. doi:10.1016/S0140-6736(18)30207-1

Lee, H. A., Chu, K. B., Moon, E. K., Kim, S. S., and Quan, F. S. (2020). Sensitization to oxidative stress and G2/M cell cycle arrest by histone deacetylase inhibition in hepatocellular carcinoma cells. *Free Radic. Biol. Med.* 147, 129–138. doi:10.1016/j.freeradbiomed.2019.12.021

Lei, Z. J., Wang, J., Xiao, H. L., Guo, Y., Wang, T., Li, Q., et al. (2015). Lysine-specific demethylase 1 promotes the stemness and chemoresistance of Lgr5⁺ liver cancer initiating cells by suppressing negative regulators of β-catenin signaling. *Oncogene* 34 (24), 3214–3298. doi:10.1038/onc.2015.182

Leung, H. W., Leung, C. O. N., Lau, E. Y., Chung, K. P. S., Mok, E. H., Lei, M. M. L., et al. (2021). EPHB2 activates β-catenin to enhance cancer stem cell properties and drive sorafenib resistance in hepatocellular carcinoma. *Cancer Res.* 81 (12), 3229–3240. doi:10.1158/0008-5472.CAN-21-0184

Li, D., Yao, Y., Rao, Y., Huang, X., Wei, L., You, Z., et al. (2022). Cholesterol sensor SCAP contributes to sorafenib resistance by regulating autophagy in hepatocellular carcinoma. *J. Exp. Clin. Cancer Res.* 41 (1), 116. doi:10.1186/s13046-022-02306-4

Li, S., Dai, W., Mo, W., Li, J., Feng, J., Wu, L., et al. (2017). By inhibiting PFKFB3, aspirin overcomes sorafenib resistance in hepatocellular carcinoma. *Int. J. Cancer* 141 (12), 2571–2584. doi:10.1002/ijc.31022

Li, T. T., Mou, J., Pan, Y. J., Huo, F. C., Du, W. Q., Liang, J., et al. (2021). MicroRNA-138-1-3p sensitizes sorafenib to hepatocellular carcinoma by targeting PAK5 mediated β-catenin/ABCB1 signaling pathway. *J. Biomed. Sci.* 28 (1), 56. doi:10.1186/s12929-021-00752-4

Li, W., Dong, X., He, C., Tan, G., Li, Z., Zhai, B., et al. (2019). LncRNA SNHG1 contributes to sorafenib resistance by activating the Akt pathway and is positively regulated by miR-21 in hepatocellular carcinoma cells. *J. Exp. Clin. Cancer Res.* 38 (1), 183. doi:10.1186/s13046-019-1177-0

Lin, Z., Xia, S., Liang, Y., Ji, L., Pan, Y., Jiang, S., et al. (2020). LXR activation potentiates sorafenib sensitivity in HCC by activating microRNA-378a transcription. *Theranostics* 10 (19), 8834–8850. doi:10.7150/thno.45158

Liu, K. L., Zhu, K., and Zhang, H. (2021). An overview of the development of EED inhibitors to disable the PRC2 function. *RSC Med. Chem.* 13 (1), 39–53. doi:10.1039/d1md00274k

Liu, W., Ning, J., Li, C., Hu, J., Meng, Q., Lu, H., et al. (2016). Overexpression of Sphk2 is associated with gefitinib resistance in non-small cell lung cancer. *Tumour Biol.* 37 (5), 6331–6336. doi:10.1007/s13277-015-4480-1

Llovet, J. M., Kelley, R. K., Villanueva, A., Singal, A. G., Pikarsky, E., Roayaie, S., et al. (2021). Hepatocellular carcinoma. *Nat. Rev. Dis. Prim.* 7 (1), 6. doi:10.1038/s41572-020-00240-3

Llovet, J. M., Montal, R., Sia, D., and Finn, R. S. (2018). Molecular therapies and precision medicine for hepatocellular carcinoma. *Nat. Rev. Clin. Oncol.* 15 (10), 599–616. doi:10.1038/s41571-018-0073-4

Llovet, J. M., Ricci, S., Mazzaferro, V., Hilgard, P., Gane, E., Blanc, J. F., et al. (2008). Sorafenib in advanced hepatocellular carcinoma. *N. Engl. J. Med.* 359 (4), 378–390. doi:10.1056/NEJMoa0708857

Lou, H., and Dean, M. (2007). Targeted therapy for cancer stem cells: The patched pathway and ABC transporters. *Oncogene* 26 (9), 1357–1360. doi:10.1038/sj.onc.1210200

Lu, S., Yao, Y., Xu, G., Zhou, C., Zhang, Y., Sun, J., et al. (2018). CD24 regulates sorafenib resistance via activating autophagy in hepatocellular carcinoma. *Cell Death Dis.* 9 (6), 646. doi:10.1038/s41419-018-0681-z

Matsui, J., Yamamoto, Y., Funahashi, Y., Tsuruoka, A., Watanabe, T., Wakabayashi, T., et al. (70802008). E7080, a novel inhibitor that targets multiple kinases, has potent antitumor activities against stem cell factor producing human small cell lung cancer H146, based on angiogenesis inhibition. *Int. J. Cancer* 122 (3), 664–671. doi:10.1002/ijc.23131

Matsuki, M., Hoshi, T., Yamamoto, Y., Ikemori-Kawada, M., Minoshima, Y., Funahashi, Y., et al. (2018). Lenvatinib inhibits angiogenesis and tumor fibroblast growth factor signaling pathways in human hepatocellular carcinoma models. *Cancer Med.* 7 (6), 2641–2653. doi:10.1002/cam4.1517

Meister, G., and Tuschl, T. (2004). Mechanisms of gene silencing by double-stranded RNA. *Nature* 431 (7006), 343–349. doi:10.1038/nature02873

Mohammad, R. M., Muqbil, I., Lowe, L., Yedjou, C., Hsu, H. Y., Lin, L. T., et al. (2015). Broad targeting of resistance to apoptosis in cancer. *Semin. Cancer Biol.* 35, S78–S103. doi:10.1016/j.semcancer.2015.03.001

Mok, E. H. K., Leung, C. O. N., Zhou, L., Lei, M. M. L., Leung, H. W., Tong, M., et al. (2022). Caspase-3-Induced activation of SREBP2 drives drug resistance via promotion of cholesterol biosynthesis in hepatocellular carcinoma. *Cancer Res.* 82 (17), 3102–3115. doi:10.1158/0008-5472.CAN-21-2934

Moosavi, F., Giovannetti, E., Peters, G. J., and Firuzi, O. (2021). Combination of HGF/MET-targeting agents and other therapeutic strategies in cancer. *Crit. Rev. Oncol. Hematol.* 160, 103234. doi:10.1016/j.critrevonc.2021.103234

Morgillo, F., Bareschino, M. A., Bianco, R., Tortora, G., and Ciardiello, F. (2007). Primary and acquired resistance to anti-EGFR targeted drugs in cancer therapy. *Differentiation* 75 (9), 788–799. doi:10.1111/j.1432-0436.2007.00200.x

Neophytou, C. M., Trougakos, I. P., Erin, N., and Papageorgis, P. (2021). Apoptosis deregulation and the development of cancer multi-drug resistance. *Cancers (Basel)* 13 (17), 4363. doi:10.3390/cancers13174363

Nieto, M. A., Huang, R. Y., Jackson, R. A., and Thiery, J. P. (2016). Emt: 2016. *Cell* 166 (1), 21–45. doi:10.1016/j.cell.2016.06.028

Nishida, N., Kitano, M., Sakurai, T., and Kudo, M. (2015). Molecular mechanism and prediction of sorafenib chemoresistance in human hepatocellular carcinoma. *Dig. Dis.* 33 (6), 771–779. doi:10.1159/000439102

Pu, W., Li, J., Zheng, Y., Shen, X., Fan, X., Zhou, J. K., et al. (2018). Targeting Pin1 by inhibitor API-1 regulates microRNA biogenesis and suppresses hepatocellular carcinoma development. *Hepatology* 68 (2), 547–560. doi:10.1002/hep.29819

Reiter, F. P., Denk, G., Ziesch, A., Ofner, A., Wimmer, R., Hohenester, S., et al. (2019). Predictors of ribociclib-mediated antitumour effects in native and sorafenib-resistant human hepatocellular carcinoma cells. *Cell Oncol. (Dordr)* 42 (5), 705–715. doi:10.1007/s13402-019-00458-8

Repana, D., and Ross, P. (2015). Targeting FGF19/FGFR4 pathway: A novel therapeutic strategy for hepatocellular carcinoma. *Diseases* 3 (4), 294–305. doi:10.3390/diseases3040294

Santoro, A., Simonelli, M., Rodriguez-Lope, C., Zucali, P., Camacho, L. H., Granito, A., et al. (2013). A Phase-1b study of tivantinib (ARQ 197) in adult patients with hepatocellular carcinoma and cirrhosis. *Br. J. Cancer* 108 (1), 21–24. doi:10.1038/bjc.2012.556

Schieber, M. S., and Chandel, N. S. (2013). ROS links glucose metabolism to breast cancer stem cell and EMT phenotype. *Cancer Cell* 23 (3), 265–267. doi:10.1016/j.ccr.2013.02.021

Schmittnaegel, M., and De Palma, M. (2017). Reprogramming tumor blood vessels for enhancing immunotherapy. *Trends Cancer* 3 (12), 809–812. doi:10.1016/j.trecan.2017.10.002

Semenza, G. L. (2013). HIF-1 mediates metabolic responses to intratumoral hypoxia and oncogenic mutations. *J. Clin. Invest.* 123 (9), 3664–3671. doi:10.1172/JCI67230

Shahar, N., and Larisch, S. (2020). Inhibiting the inhibitors: Targeting anti-apoptotic proteins in cancer and therapy resistance. *Drug Resist Updat* 52, 100712. doi:10.1016/j.drup.2020.100712

Shang, R., Song, X., Wang, P., Zhou, Y., Lu, X., Wang, J., et al. (2021). Cabozantinib-based combination therapy for the treatment of hepatocellular carcinoma. *Gut* 70 (9), 1746–1757. doi:10.1136/gutjnl-2020-320716

Shen, Y. A., Wang, C. Y., Hsieh, Y. T., Chen, Y. J., and Wei, Y. H. (2015). Metabolic reprogramming orchestrates cancer stem cell properties in nasopharyngeal carcinoma. *Cell Cycle* 14 (1), 86–98. doi:10.4161/15384101.2014.974419

Shen, Y. C., Ou, D. L., Hsu, C., Lin, K. L., Chang, C. Y., Lin, C. Y., et al. (2013). Activating oxidative phosphorylation by a pyruvate dehydrogenase kinase inhibitor overcomes sorafenib resistance of hepatocellular carcinoma. *Br. J. Cancer* 108 (1), 72–81. doi:10.1038/bjc.2012.559

Shi, T., Iwama, H., Fujita, K., Kobara, H., Nishiyama, N., Fujihara, S., et al. (2021). Evaluating the effect of lenvatinib on sorafenib-resistant hepatocellular carcinoma cells. *Int. J. Mol. Sci.* 22 (23), 13071. doi:10.3390/ijms222313071

Shi, W., Zhang, S., Ma, D., Yan, D., Zhang, G., Cao, Y., et al. (2020). Targeting SphK2 reverses acquired resistance of regorafenib in hepatocellular carcinoma. *Front. Oncol.* 10, 694. doi:10.3389/fonc.2020.00694

Stine, Z. E., Schug, Z. T., Salvino, J. M., and Dang, C. V. (2022). Targeting cancer metabolism in the era of precision oncology. *Nat. Rev. Drug Discov.* 21, 141–162. doi:10.1038/s41573-021-00339-6

Su, M., Mei, Y., and Sinha, S. (2013). Role of the crosstalk between autophagy and apoptosis in cancer. *J. Oncol.* 2013, 102735. doi:10.1155/2013/102735

Sun, K., and Lai, E. C. (2013). Adult-specific functions of animal microRNAs. *Nat. Rev. Genet.* 14 (8), 535–548. doi:10.1038/nrg3471

Suski, J. M., Braun, M., Strmiska, V., and Sicinski, P. (2021). Targeting cell-cycle machinery in cancer. *Cancer Cell* 39 (6), 759–778. doi:10.1016/j.ccell.2021.03.010

Tan, W., Luo, X., Li, W., Zhong, J., Cao, J., Zhu, S., et al. (2019). TNF-α is a potential therapeutic target to overcome sorafenib resistance in hepatocellular carcinoma. *EBioMedicine* 40, 446–456. doi:10.1016/j.ebiom.2018.12.047

Tang, W., Chen, Z., Zhang, W., Cheng, Y., Zhang, B., Wu, F., et al. (2020). The mechanisms of sorafenib resistance in hepatocellular carcinoma: Theoretical basis and therapeutic aspects. *Signal Transduct. Target Ther.* 5 (1), 87. doi:10.1038/s41392-020-0187-x

Tong, M., Che, N., Zhou, L., Luk, S. T., Kau, P. W., Chai, S., et al. (2018). Efficacy of annexin A3 blockade in sensitizing hepatocellular carcinoma to sorafenib and regorafenib. *J. Hepatol.* 69 (4), 826–839. doi:10.1016/j.jhep.2018.05.034

Tovar, V., Cornella, H., Moeini, A., Vidal, S., Hoshida, Y., Sia, D., et al. (2017). Tumour initiating cells and IGF/FGF signalling contribute to sorafenib resistance in hepatocellular carcinoma. *Gut* 66 (3), 530–540. doi:10.1136/gutjnl-2015-309501

Vakil, V., and Trappe, W. (2021). Dosage strategies for delaying resistance emergence in heterogeneous tumors. *FEBS Open Bio* 11 (5), 1322–1331. doi:10.1002/2211-5463.13129

Vakil, V., and Trappe, W. (2022). Drug-resistant cancer treatment strategies based on the dynamics of clonal evolution and PKPD modeling of drug combinations. *IEEE/ACM Trans. Comput. Biol. Bioinform.* 19 (3), 1603–1614. doi:10.1109/TCBB.2020.3045315

Venepalli, N. K., and Goff, L. (2013). Targeting the HGF-cMET Axis in hepatocellular carcinoma. *Int. J. Hepatol.* 2013, 341636. doi:10.1155/2013/341636

Vidal, S. J., Rodriguez-Bravo, V., Galsky, M., Cordon-Cardo, C., and Domingo-Domenech, J. (2014). Targeting cancer stem cells to suppress acquired chemotherapy resistance. *Oncogene* 33 (36), 4451–4463. doi:10.1038/onc.2013.411

Villanueva, A. (2019). Hepatocellular carcinoma. *N. Engl. J. Med.* 380 (15), 1450–1462. doi:10.1056/NEJMra1713263

Vishnoi, K., Ke, R., Viswakarma, N., Srivastava, P., Kumar, S., Das, S., et al. (2022). Ets1 mediates sorafenib resistance by regulating mitochondrial ROS pathway in hepatocellular carcinoma. *Cell Death Dis.* 13 (7), 581. doi:10.1038/s41419-022-05022-1

Wang, J., Zhang, N., Han, Q., Lu, W., Wang, L., Yang, D., et al. (2019). Pin1 inhibition reverses the acquired resistance of human hepatocellular carcinoma cells to Regorafenib via the Gli1/Snail/E-cadherin pathway. *Cancer Lett.* 444, 82–93. doi:10.1016/j.canlet.2018.12.010

Wang, Z., Zhu, Q., Li, X., Ren, X., Li, J., Zhang, Y., et al. (2022). TOP2A inhibition reverses drug resistance of hepatocellular carcinoma to regorafenib. *Am. J. Cancer Res.* 12 (9), 4343–4360. PMID: 36225636; PMCID: PMC9548008.

Wei, L., Wang, X., Lv, L., Liu, J., Xing, H., Song, Y., et al. (2019). The emerging role of microRNAs and long noncoding RNAs in drug resistance of hepatocellular carcinoma. *Mol. Cancer* 18 (1), 147. doi:10.1186/s12943-019-1086-z

Wei, S., Kozono, S., Kats, L., Nechama, M., Li, W., Guarnerio, J., et al. (2015). Active Pin1 is a key target of all-trans retinoic acid in acute promyelocytic leukemia and breast cancer. *Nat. Med.* 21 (5), 457–466. doi:10.1038/nm.3839

Xin, H. W., Ambe, C. M., Hari, D. M., Wiegand, G. W., Miller, T. C., Chen, J. Q., et al. (2013). Label-retaining liver cancer cells are relatively resistant to sorafenib. *Gut* 62 (12), 1777–1786. doi:10.1136/gutjnl-2012-303261

Xu, Y., Huang, J., Ma, L., Shan, J., Shen, J., Yang, Z., et al. (2016). MicroRNA-122 confers sorafenib resistance to hepatocellular carcinoma cells by targeting IGF-1R to regulate RAS/RAF/ERK signaling pathways. *Cancer Lett.* 371 (2), 171–181. doi:10.1016/j.canlet.2015.11.034

Yakes, F. M., Chen, J., Tan, J., Yamaguchi, K., Shi, Y., Yu, P., et al. (2011). Cabozantinib (XL184), a novel MET and VEGFR2 inhibitor, simultaneously suppresses metastasis, angiogenesis, and tumor growth. *Mol. Cancer Ther.* 10 (12), 2298–2308. doi:10.1158/1535-7163.MCT-11-0264

Yarden, Y., and Pines, G. (2012). The ERBB network: At last, cancer therapy meets systems biology. *Nat. Rev. Cancer* 12 (8), 553–563. doi:10.1038/nrc3309

Yazdani, H. O., Huang, H., and Tsung, A. (2019). Autophagy: Dual response in the development of hepatocellular carcinoma. *Cells* 8 (2), 91. doi:10.3390/cells8020091

Yu, T., Yu, J., Lu, L., Zhang, Y., Zhou, Y., Zhou, Y., et al. (2021). MT1JP-mediated miR-24-3p/BCL2L2 axis promotes Lenvatinib resistance in hepatocellular carcinoma cells by inhibiting apoptosis. *Cell Oncol. (Dordr).* 44 (4), 821–834. doi:10.1007/s13402-021-00605-0

Yu, Z., Feng, H., Zhuo, Y., Li, M., Zhu, X., Huang, L., et al. (2020). Bufalin inhibits Hepatitis B virus-associated hepatocellular carcinoma development through androgen receptor dephosphorylation and cell cycle-related kinase degradation. *Cell Oncol. (Dordr).* 43 (6), 1129–1145. doi:10.1007/s13402-020-00546-0

Zhai, B., Hu, F., Jiang, X., Xu, J., Zhao, D., Liu, B., et al. (2014). Inhibition of Akt reverses the acquired resistance to sorafenib by switching protective autophagy to autophagic cell death in hepatocellular carcinoma. *Mol. Cancer Ther.* 13 (6), 1589–1598. doi:10.1158/1535-7163.MCT-13-1043

Zhang, P., Sun, H., Wen, P., Wang, Y., Cui, Y., and Wu, J. (2021). circRNA circMED27 acts as a prognostic factor and mediator to promote lenvatinib resistance of hepatocellular carcinoma. *Mol. Ther. Nucleic Acids* 27, 293–303. doi:10.1016/j.omtn.2021.12.001

Zhang, X., He, B., Chen, E., Lu, J., Wang, J., Cao, H., et al. (2021). The aryl hydrocarbon receptor ligand ITE inhibits cell proliferation and migration and enhances sensitivity to drug-resistance in hepatocellular carcinoma. *J. Cell Physiol.* 236 (1), 178–192. doi:10.1002/jcp.29832

Zhang, X., Wu, L., Xu, Y., Yu, H., Chen, Y., Zhao, H., et al. (2020). Microbiota-derived SSL6 enhances the sensitivity of hepatocellular carcinoma to sorafenib by down-regulating glycolysis. *Cancer Lett.* 481, 32–44. doi:10.1016/j.canlet.2020.03.027

Zhao, H., Cheng, X., Yu, J., and Li, Y. (2021). Stabilization of snail maintains the sorafenib resistance of hepatocellular carcinoma cells. *Arch. Biochem. Biophys.* 699, 108754. doi:10.1016/j.abb.2021.108754

Zhao, Y. G., Codogno, P., and Zhang, H. (2021). Machinery, regulation and pathophysiological implications of autophagosome maturation. *Nat. Rev. Mol. Cell Biol.* 22 (11), 733–750. doi:10.1038/s41580-021-00392-4

Zhao, Z., Song, J., Zhang, D., Wu, F., Tu, J., and Ji, J. (2021). Oxysophocarpine suppresses FGFR1-overexpressed hepatocellular carcinoma growth and sensitizes the therapeutic effect of lenvatinib. *Life Sci.* 264, 118642. doi:10.1016/j.lfs.2020.118642

Zhao, Z., Zhang, D., Wu, F., Tu, J., Song, J., Xu, M., et al. (2021). Sophoridine suppresses lenvatinib-resistant hepatocellular carcinoma growth by inhibiting RAS/MEK/ERK axis via decreasing VEGFR2 expression. *J. Cell Mol. Med.* 25 (1), 549–560. doi:10.1111/jcmm.16108

Zhong, Z., Sanchez-Lopez, E., and Karin, M. (2016). Autophagy, inflammation, and immunity: A troika governing cancer and its treatment. *Cell* 166 (2), 288–298. doi:10.1016/j.cell.2016.05.051
